# Genome-wide proteomic profiling reveals the role of dominance protein expression in heterosis in immature maize ears

**DOI:** 10.1038/s41598-017-15985-3

**Published:** 2017-11-23

**Authors:** Xiaojiao Hu, Hongwu Wang, Kun Li, Yujin Wu, Zhifang Liu, Changling Huang

**Affiliations:** Institute of Crop Science, Chinese Academy of Agricultural Sciences, National Engineering Laboratory for Crop Molecular Breeding, Beijing, 100081 China

## Abstract

Heterosis refers to the phenomenon in which hybrid progeny show superior performance relative to their parents. Early maize ear development shows strong heterosis in ear architecture traits and greatly affects grain yield. To explore the underlying molecular mechanisms, genome-wide proteomics of immature ears of maize hybrid ZD909 and its parents were analyzed using tandem mass tag (TMT) technology. A total of 9,713 proteins were identified in all three genotypes. Among them, 3,752 (38.6%) proteins were differentially expressed between ZD909 and its parents. Multiple modes of protein action were discovered in the hybrid, while dominance expression patterns accounted for 63.6% of the total differentially expressed proteins (DEPs). Protein pathway enrichment analysis revealed that high parent dominance proteins mainly participated in carbon metabolism and nitrogen assimilation processes. Our results suggested that the dominant expression of favorable alleles related to C/N metabolism in the hybrid may be essential for ZD909 ear growth and heterosis formation. Integrated analysis of proteomic and quantitative trait locus (QTL) data further support our DEP identification and provide useful information for the discovery of genes associated with ear development. Our study provides comprehensive insight into the molecular mechanisms underlying heterosis in immature maize ears from a proteomic perspective.

## Introduction

Heterosis, or hybrid vigor, refers to the phenomenon in which hybrid progeny show superior performance in biomass, yield, or other agronomic traits compared with that of their parents^1,2^. The exploitation of heterosis has revolutionized crop breeding and production worldwide. Maize is the most successful crop utilizing hybrid vigor. Today, 65% of maize acreage worldwide is planted with hybrid varieties^3,4^. In China, hybrids cover 97% of the total maize area and have contributed 40% of the grain yield increase^5^. Given its biological and economic importance, the genetic mechanisms of heterosis have fascinated scientists for over a century. The major genetic models proposed to explain heterosis are dominance, overdominance, and epistasis^6–8^. Quantitative trait locus (QTL) mapping studies of yield-related traits in various plant species have suggested that all three models are involved in the manifestation of heterosis^9–12^. However, these models are not connected with molecular principles and none of these models own can adequately explains the complexity of heterosis.

In recent years, the rapid development of molecular biology techniques has revealed that genome-wide changes at the genome, transcriptome, epigenome, and proteome levels may greatly contribute to heterosis. Next-generation sequencing has shown that sequence variations such as single-nucleotide polymorphisms (SNPs) and small indels, as well as larger structural variations such as copy number variation (CNV) and presence and absence variation (PAV), may greatly contribute to phenotypic diversity in many important crops^13,14^. In maize, over 10% of genes in the B73 reference genome exhibit CNV/PAV among genotypes^15,16^. High levels of variability in gene content among maize inbred lines will result in hybrids with more genes than either inbred parent, which might lead to significantly increased performance in hybrid plants. At the transcriptional level, genome-wide expression profiling studies have revealed substantial changes in gene expression among hybrids and their parents in many species^17–23^. All possible differential gene expression patterns have been discovered in hybrids, including additivity, high- and low-parent dominance, underdominance, and overdominance^18,24^. Studies have revealed that additive gene expression was prevalent and positively correlated with heterosis and high yield in many species^21,25,26^. However, other studies suggest that hybrid expression patterns outside of the parental range may indicate novel regulation and result in superior performance^27,28^. This discrepancy may depend on differences in species, parental genotypes, tissues, and developmental stages, as there is also variation in the relative level of heterosis for traits among different hybrids. In addition to these findings, a number of studies support potential links between epigenetic regulation and heterosis. DNA methylation, histone modification, and small RNAs can alter gene expression and contribute to heterotic effects in hybrids^20,29,30^.

As changes at the level of mRNA may not always reflect the changes in the level of protein, gene expression variation among hybrids and parents have also been examined at the proteome level^31^. In recent decades, 2-D gel electrophoresis (2-DE) combined with mass spectrometry (MS) has been widely utilized to detect correlations between differential protein expression and hybrid vigor in various tissues of wheat, maize, rice, and other species^32–35^. A 2-DE study of the wheat root proteome showed that hybridization can result in expression differences between hybrids and parents^35^. Comparative proteomic analysis of 25- and 35-day-old immature maize embryos revealed that 141 of 597 proteins exhibited nonadditive accumulation in at least one hybrid, and these proteins were mainly involved in development, protein metabolism, redox regulation, glycolysis, and amino acid metabolism^36^. In dry and 24 h imbibed maize seed embryos, 134 and 191 differentially expressed proteins were discovered between the hybrid and its parents, respectively, among which 47.01% and 34.55% protein spots displayed nonadditively expressed patterns^37^. Comparative leaf proteomic analysis between the super hybrid rice LYP9 and its parents at tillering, flowering, and grain-filling stages revealed that 36.6–46.2% of differentially expressed proteins in the hybrid showed nonadditively expressed patterns^34^. Although the gel base method has successfully identified heterosis-related proteins in many species, it has clear drawbacks, including limited resolution and restricted sample throughput. In recent years, with the development of proteomic quantitative methods, label-based and label-free MS-based approaches have provided more precision and an efficient way to quantify multiple samples in parallel^38–40^. Tandem mass tags (TMT) and isobaric tags for relative and absolute quantification (iTRAQ) are two typical isobaric labeling reagents developed for MS-based protein detection and quantification^41–43^. For instance, TMT has been successfully applied to conduct a quantitative proteomics study of wheat seeds during artificial aging and priming and to detect alterations in development and photosynthesis-related proteins in diploid and triploid rice^41,44^.

Immature maize ear development is an important and complex process. Significant hybrid vigor has been observed in maize ear architecture traits, such as ear length, ear diameter, kernel row number, and kernel number per row, which contribute greatly to maize yield^45^. ZD909 is an excellent maize hybrid bred by our research group and approved by the National Crop Variety Approval Committee of China in 2011. The variety exhibits strong heterosis in ear architectural traits, with large ears and high yields, and is an excellent material for molecular dissection of heterosis manifestation during immature maize ear development. In this study, we conducted genome-wide proteomic profiling of immature ears of ZD909 and its parent lines Z58 and HD568 by MS/MS using tandem mass tag technology. Large numbers of differentially expressed proteins between the hybrid and its parents were identified. Parental expression-level dominance proteins, which are mainly involved in carbohydrate and nitrogen metabolism, account for the majority of differentially expressed proteins and may greatly contribute to maize ear heterosis. The molecular insights provided by this study might help to better understand the possible molecular networks involved in maize heterosis at the proteome level.

## Results

### Heterosis in ear traits of maize hybrid ZD909

To explore heterosis manifestation of maize immature ear development, the uppermost ears at 14-leaf stage (V14) were collected form maize hybrid ZD909 and its parents Z58 and HD568. The V14 stage occurs approximately two weeks before flowering when the immature ears are undergoing floral organs differentiation with an increase in ear size and the rapid elongation of pistils. The number of ovules on an ear and size of ear are being determined, and the young ear serves as a critical sink, providing nutrients for bearing kernels during the reproductive stage. This development stage has been reported to be crucial to heterosis of dry matter accumulation and grain yield in maize^46,47^.

**Figure 1:**
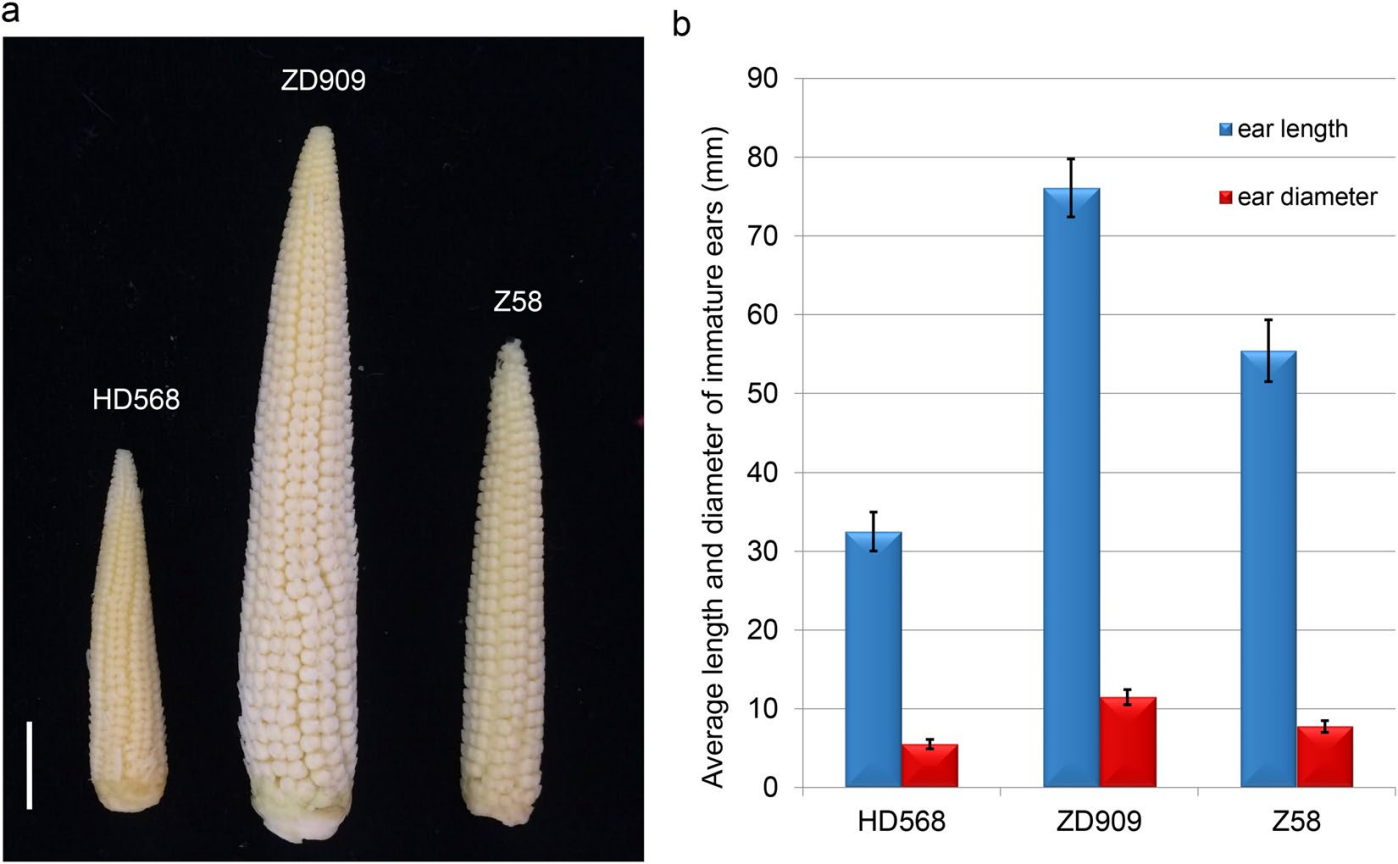
Characterization of the immature ear traits of maize hybrid ZD909 and its parental lines. (**a**) Phenotypic differences of immature ears of ZD909, Z58 and HD568. The scale bar indicates 1 cm. (**b**) Ear length and diameter of ZD909, Z58 and HD568. Error bars indicate standard error.

At V14 stage, the maize hybrid ZD909 exhibits strong heterosis in ear architectural traits. Immature ears of ZD909 are more vigorous than its parents HD568 and Z58 in terms of ear length and ear diameter, and the ears of the maternal line Z58 are larger than in HD568 (Fig. 1 and Table 1). We further calculated the midparent heterosis (MPH) and best parent heterosis (BPH) of immature ear traits. Significant (p < 0.01) MPH and BPH were observed for ear length and ear diameter, with higher values for ear diameter. Significant heterosis was also discovered for ear length, ear diameter, ear row number, and kernel number per row of ZD909 mature ears. The MPH and BPH values for these traits varied from 21.42% to 66.29% and from 18.18% to 65.23%, respectively (Table 1). The results suggested that ZD909 is a useful material with which to investigate the molecular mechanisms underlying maize ear heterosis.

**Table 1:** Heterosis analysis of ear architectural traits of ZD909.

Stages	Traits^a^	Z58	HD568	ZD909	MPH (100%)	BPH (100%)
immature ear	EL (mm)	55.42 ± 3.92	32.50 ± 2.47	76.08 ± 3.68	26.07*	37.29*
	ED(mm)	7.74 ± 0.75	5.49 ± 0.60	11.47 ± 0.97	73.31*	48.15*
mature ear	EL(mm)	143.5 ± 10.26	133.57 ± 17.10	192.71 ± 6.75	39.11*	34.29*
	ED (mm)	42.91 ± 3.59	40.61 ± 3.83	50.70 ± 1.83	21.42*	18.18*
	ERN	11.5 ± 0.53	13.2 ± 0.97	16.2 ± 1.40	31.06*	22.52*
	KNPR	24.87 ± 1.81	24.56 ± 3.24	41.1 ± 1.85	66.29*	65.23*

^a^EL, ear length; ED, ear diameter; ERN, ear row number; KNPR, Kernel number per row. Values are means ± standard deviation based on 20 plants. The Student’s t-test was used for statistical analyses (*indicates p-values < 0.01). MPH, Midparent heterosis; BPH, Best parent heterosis.

### Protein identification and quantification by mass spectrometry

To investigate heterosis manifestation at early stages of maize ear development, we conducted proteomic profiling of immature ears of ZD909 and its parents Z58 and HD568 by MS/MS using TMT technology. Total soluble proteins of three independent biological replicates of each genotype were digested and isobarically labeled using TMT. The labeled peptides were subjected to LC-MS/MS analysis. Raw mass data were processed and searched against the NCBI’s RefSeq protein sequence database of *Zea mays* (taxid: 4577) using Proteome Discoverer software. In total, 10,265 proteins were identified from 66,226 distinct peptides with at least one unique peptide. For subsequent analyses, only 9,713 proteins, quantified in all three genotypes, were considered. The complete peptide and protein match information of the 9,713 proteins is provided in Supplementary Table S1.

### Protein expression divergence between ZD909 and its parents

**Figure 2:**
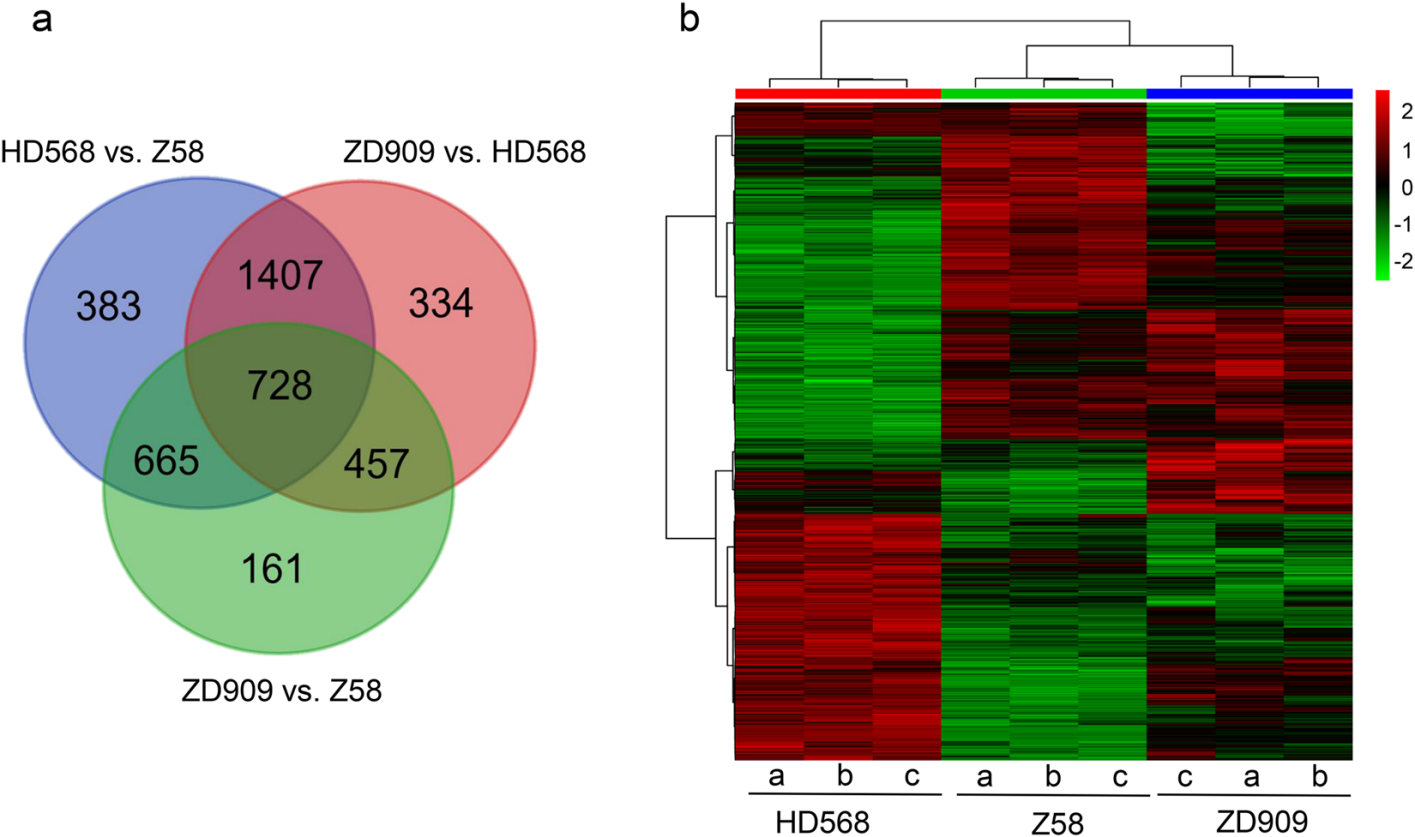
Venn diagram comparison and hierarchical cluster analysis of differentially expressed proteins among genotypes. (**a**) Venn diagram comparison of differential expressed proteins between the hybrid and its parents. (**b**) Hierarchical cluster analysis of differentially expressed proteins among genotypes. The color key represents protein expression quantity. Red indicates high relative expression and green indicates low relative expression. a, b, c indicate three biological replicates.

To identify differentially expressed proteins (DEPs) between ZD909 and parental inbred lines, one-way ANOVA followed by t-tests with a Bonferroni correction was performed on quantitative values for the identified protein. When controlling the false discovery rate at 0.05, 4,135 proteins were present in significantly different quantities across the three genotypes, accounting for 42.6% of analyzed proteins. Among them, 3,183 proteins were differentially expressed between HD568 and Z58, with 1,530 upregulated and 1,653 downregulated proteins (Table 2). The high expression divergence suggested a large genetic distance between the two parental lines. Comparisons between ZD909 and two parent lines revealed 3,752 DEPs, which is more than the DEPs between the two parents. In all, 2,926 proteins were differentially expressed between ZD909 and HD568, including 1,530 upregulated and 1,396 downregulated proteins, and 2,011 differentially expressed proteins were identified between ZD909 and Z58, including 1,010 upregulated and 1,001 downregulated proteins (Table 2). Venn diagram analysis revealed that 67.1% (1,407 and 728) of DEPs between HD568 and Z58 were differentially expressed between ZD909 and HD568, whereas fewer DEPs (43.8%, 728 and 665) were differentially expressed between ZD909 and Z58. Only 22.9% (728 of 3,183) of DEPs were shared by the three comparisons (Fig. 2a). These results show that protein expression variation is prevalent but differs among the three genotypes. We further investigated the profile changes of differentially expressed proteins among the three genotypes using hierarchical cluster analysis (Fig. 2b). The results suggest that protein expression patterns in ZD909 are more similar to the maternal parent Z58 than to the paternal parent HD568.

**Table 2:** The differentially expressed proteins between ZD909 and the parents.

Comparison Groups	Up		Down		Total	
	Number	Percentage	Number	Percentage	Number	Percentage
HD568 vs Z58	1,530	15.8%	1,653	17.0%	3,183	32.8%
ZD909 vs HD568	1,530	15.8%	1,396	14.4%	2,926	30.2%
ZD909 vs Z58	1,001	10.4%	1,010	10.3%	2,011	20.7%

Total analyzed genes: 9,713.

### Identification of differential protein expression patterns in hybrid

**Figure 3:**
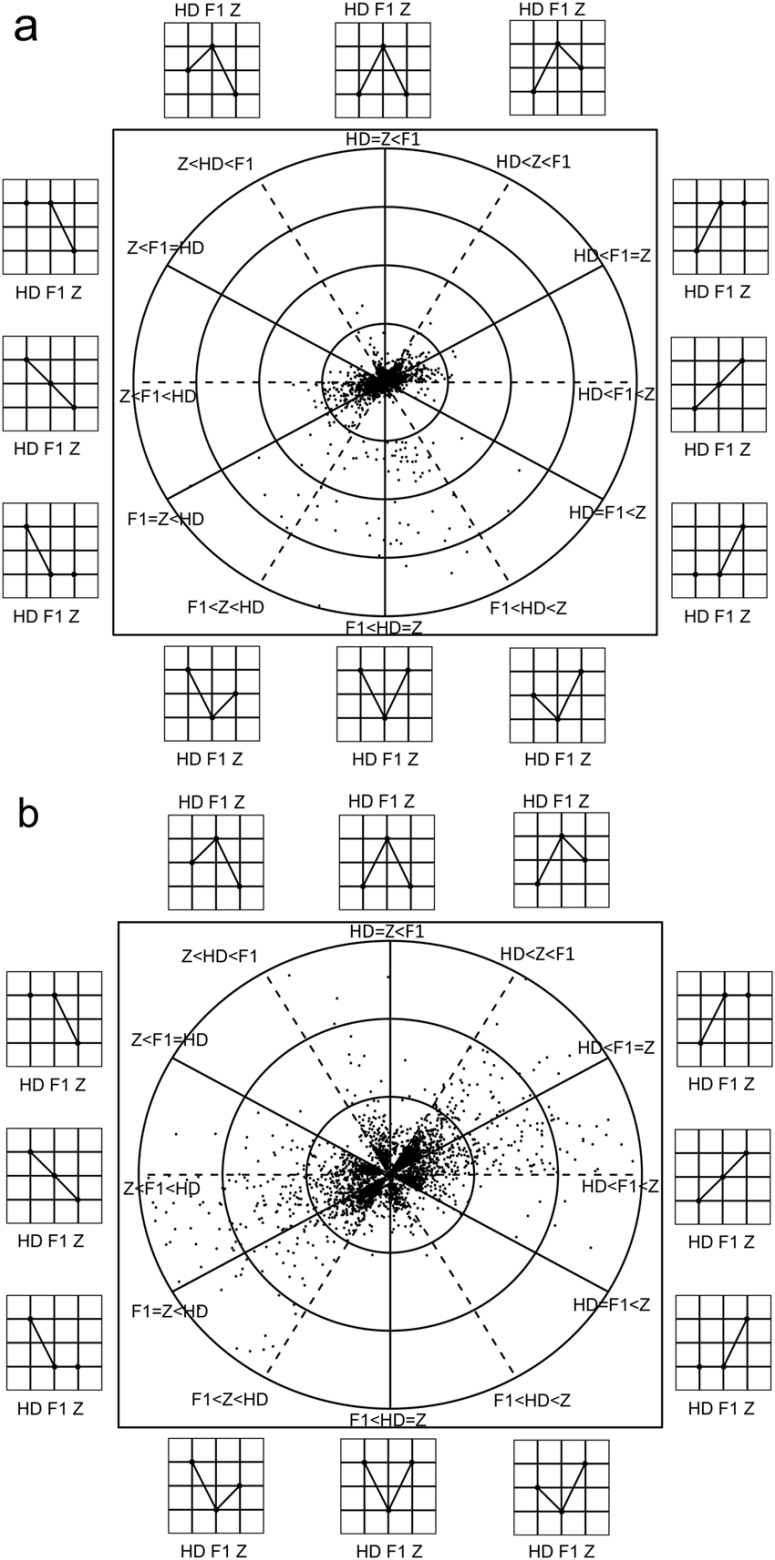
2-D presentations of expression and fold changes of differentially expressed proteins among ZD909, HD568 and Z58. HD, F1, and Z stand for HD568, ZD909, and Z58, respectively. Twelve different expression patterns are indicated clock-wise outside the plot. The radius at which a gene is plotted represents log2 fold changes between the highest and lowest expression levels among the three genotypes. The angle at which a gene is plotted represents the relationships among the means of the three genotypes. Proteins falling on the horizontal and vertical lines exhibit pure additivity and over- (or under-) dominance, respectively. Proteins plotted between lines exhibit a mode of gene action that is intermediate to that indicated by the two nearest lines. (**a**) Presentation of differentially expressed proteins with log_2_ fold changes range from 0 to 12. The four circles represent log2 fold changes of 3, 6, 9 and 12, respectively. (**b**) Presentation of differentially expressed proteins with log_2_ fold changes range from 0 to 3. The three circles represent log2 fold changes of 1, 2 and 3, respectively.

In a further investigation of differential protein expression patterns, 12 classes were defined based on a similar classification published previously^18,24^. We present our results in a 2-D concentric circle, where protein expression patterns and log2-fold changes of DEPs are plotted (Fig. 3a and b). After the removal of proteins showing expression patterns with no clear biological interpretation (‘ambiguous’ patterns), the distribution of 3,258 remaining DEPs fell into four main categories: additive, overdominance, underdominance, and parental expression-level dominance (ELD) (Supplementary Table S2). The additive category covers regions from 2 to 4 o’clock and 8 to 10 o’clock, where 560 proteins in the hybrid exhibiting expression levels between the two parents were plotted. In the overdominance category, 322 proteins distributed from 11 to 1 o’clock showed expression levels significantly higher than that in the two parents. In the underdominance category, 303 proteins within the 5 to 7 o’clock region exhibited significantly lower expression levels than in the two parents. Parental ELD was the largest category, with 2,073 DEPs, accounting for 63.6% of the total analyzed proteins. Among them, 333 and 332 proteins plotted on the 2 and 8 o’clock solid line, respectively, were categorized as HD568 dominance, and 661 and 747 proteins, falling on the 4 and 10 o’clock solid line, respectively, were categorized as Z58 dominance. Further sub-classification of the ELD proteins revealed that 1,080 proteins exhibited high parent dominance (HPD) and 993 exhibited low parent dominance (LPD) (Supplementary Table S2). These results show that a large proportion of DEPs (2,698 of 3,258, 82.8%) in ZD909 exhibited nonadditive expression patterns, which may contribute greatly to heterosis in the immature ear. The higher number of Z58 dominance proteins further confirmed that the similarity between ZD909 and Z58 was greater than that between ZD909 and HD568.

AS the critical important reproductive tissue, the maize ear inflorescence has been extensively investigated by lots of studies. Many genes controlling maize ear growth and development are now cloned, providing an emerging molecular framework for the developmental pathways. In our study, 18 well-known genes related to ear architecture development were found to be expressed in immature ears of all three genotypes, including *RA1, TSH4, FEA2, TD1, TLS1* and *KN1*, which affect the determinacy and identity of meristems*; MADS8, ZAG1, ZAG2, ZAG3 (BDE1), IFA1* and *ZFL1*, which control the floral meristem identities and floral organ specification^48,49^ (Supplementary Table S3). Further investigation revealed that 8 genes were differentially expressed between hybrid ZD909 and its parent lines. Two MADS-box genes, *ZAG1* and *ZAG2*, which are expressed in stamen and pistil and contribute to ovule development^50^, were found to be expressed in overdominance pattern in hybrid. *TLS1*, which mediates the transport of boron for vegetative and reproductive meristem development was also expressed in overdominance pattern^51^. *MADS14* and *IFA1*, which are required for determinacy of the floral meristem^52,53^, were found to be expressed at the HPD-HD568 level in ZD909 (Supplementary Table S3). Active expression of these genes, which are correlated to floral meristem determinacy and organ development, may promote the transition from vegetative to reproductive growth in hybrid, resulting in rapid growth and superior performance of hybrid ear.

### Kyoto Encyclopedia of Genes and Genomes (KEGG) pathway enrichment for nonadditively expressed proteins

**Figure 4:**
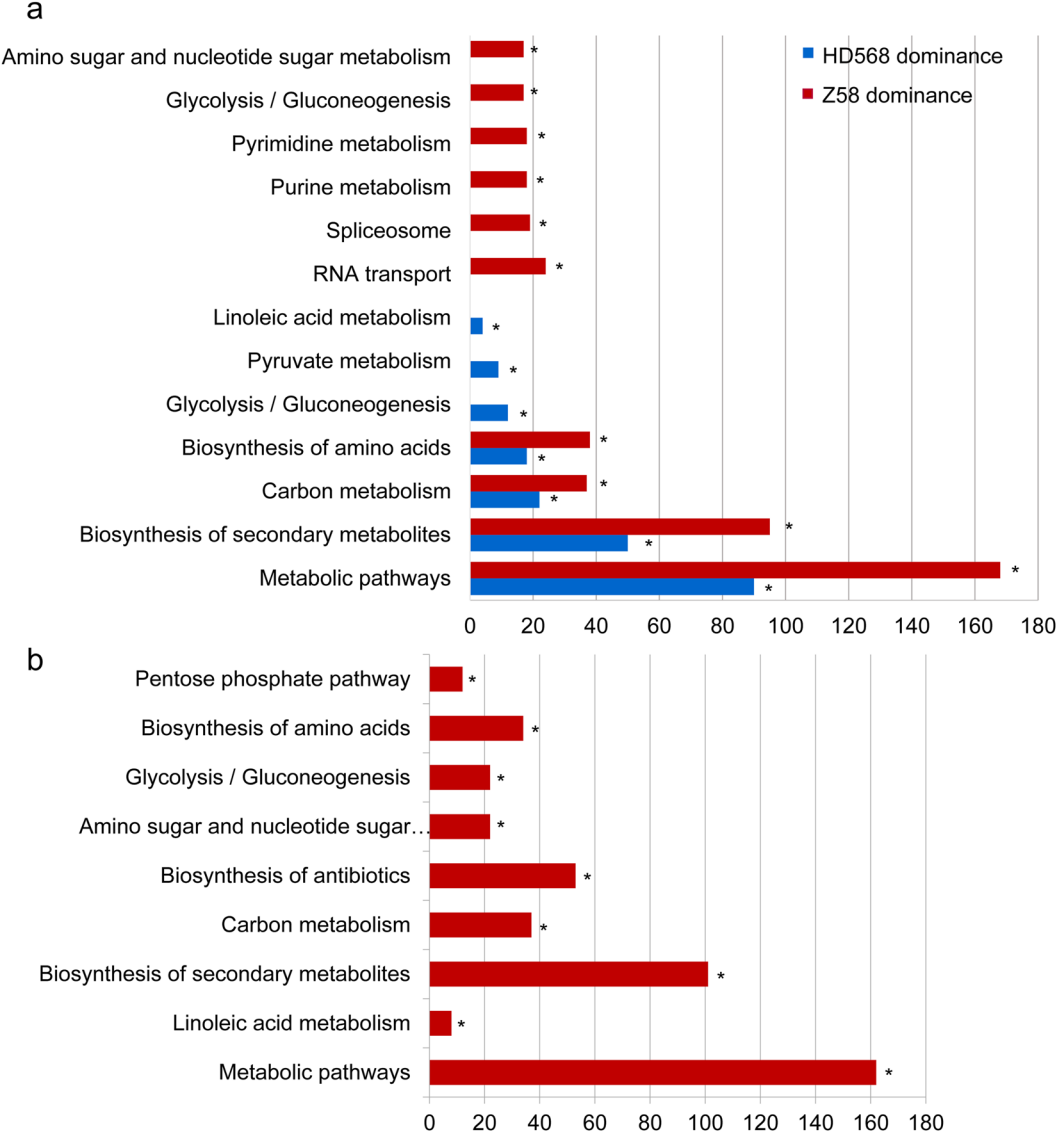
KEGG pathway enrichment analysis of parental expression level dominance (ELD) proteins. (**a**) KEGG pathway enrichment analysis of HD568 dominance and Z58 dominance proteins. (**b**) KEGG pathway enrichment analysis of high parent dominance proteins. *Indicates Benjamini-Yekutieli corrected p-value < 0.01.

There is evidence that nonadditive expression can confer novel or superior phenotypes in hybrids^27,28,32^. To understand the biological roles of DEPs with nonadditive expression patterns, we conducted KEGG pathway enrichment analysis using KOBS 3.0. For overdominance proteins, ‘metabolic pathways’ (11.2%) and ‘biosynthesis of secondary metabolites’ (7.5%) pathways were significantly overrepresented (Supplementary Tables S4 and S6). Under the ‘metabolic pathways’, the proteins related to carbon and amino acid metabolism, such as ATP synthase, pyruvate kinase, glucose-6-phosphate dehydrogenase, phosphofructokinase, Glutamate synthase, and glutamate dehydrogenase were found to be expressed at 1.2 to 5.9-fold higher in hybrid than in its parents. For underdominance proteins, only ‘ribosome biogenesis in eukaryotes’ (2.6%) was significantly overrepresented (Supplementary Tables S4 and S7). The enriched pathways were largely the same in HD568 dominance and Z58 dominance proteins, including ‘metabolic pathways’ (13.5% and 11.9%, respectively), ‘biosynthesis of secondary metabolites’ (7.5% and 6.7%), ‘carbon metabolism’ (3.3% and 2.6%), and ‘biosynthesis of amino acids’ (2.7% and 2.7%) (Fig. 4a and Supplementary Table S4). Investigation of the subclasses of ELD proteins revealed that nine pathways mainly including ‘metabolic pathways’ (15.2%), ‘biosynthesis of secondary metabolites’ (9.5%), ‘carbon metabolism’ (3.4%), ‘glycolysis/gluconeogenesis’ (2.0%), ‘biosynthesis of amino acids’ (3.1%) were significantly overrepresented in high parent dominance proteins, and no significantly enriched pathway could be detected in low parent dominance proteins (Fig. 4b and Supplementary Table S5).

Further check in detail the functions of high parent dominance proteins enriched in ‘metabolism pathway’ revealed that DEPs were mainly distributed in carbohydrate and energy metabolism, amino acid and nitrogen metabolism, lipid metabolism, and hormone metabolism (Supplementary Table S5). For carbohydrate and energy metabolism, 6 DEPs related to photosynthesis and the calvin cycle (e.g., fructose-bisphosphate aldolase and ribulose bisphosphate carboxylase), 25 DEPs related to glycolysis and gluconeogenesis (e.g., phosphofructokinase, hexokinase, and glucose-6-phosphate isomerase), 12 DEPs related to pentose phosphate pathways (e.g., phosphoglucomutase and ribulose-phosphate 3-epimerase), and 8 DEPs in the citrate or tricarboxylic acid cycle (e.g., pyruvate dehydrogenase and malate dehydrogenase) were expressed at HD568 or Z58 expression levels. For amino acid and nitrogen metabolism, 36 DEPs, including glutamine synthetase, glutamate dehydrogenase, and aminotransferase, were expressed at HD568 or Z58 expression levels (Supplementary Table S5). These results suggest that high parent dominance proteins play important roles in carbon/nitrogen metabolism. The active carbon metabolism and nitrogen assimilation in hybrid ZD909 may be essential for maize ear growth and grain yield formation. In addition, 16 DEPs related to lipid metabolism, such as long-chain-fatty-acid-CoA ligase, diacylglycerol kinase, phospholipase D, 3-hydroxyacyl-CoA dehydrogenase proteins, 4 DEPs participating in jasmonate synthesis-degradation and 10 DEPs annotated as peroxidases, were expressed at HD568 or Z58 expression levels (Supplementary Table S5). These proteins may function in plant responses to biotic and abiotic stimuli and cell signaling, which suggests that high parent dominance proteins also contribute to maize ear development with respect to stress resistance and signal transduction.

### Mapping DEPs to ear trait- and kernel trait-related QTL

Phenotypic variation for quantitative traits results from segregation at multiple QTL. In our previous studies, we mapped QTL for ear architectural traits and kernel-related traits with a recombination inbred line (RIL) mapping population with 220 families from a cross between the inbred lines Z58 and HD568^54^. The experiment was conducted under three different plant densities (52,500, 67,500, and 82,500 plant∙hm^−2^) in three environments. With the BLUP values estimated by SAS software, 42 QTL for ear length (EL), ear diameter (ED), ear row number (ERN), and kernel number per row (KNPR) and 26 QTL for hundred-kernel weight (HKW), kernel length (KL), kernel thickness (KT), and kernel width (KW) were detected under three planting densities.

To survey the relationship between protein expression variation and phenotypic changes in hybrid ZD909, we mapped DEPs from our proteomic results to these 68 QTL. In total, we mapped 3,656 of 3,752 DEPs to maize chromosomes. Of the 3,656 DEPs, 265 and 179 DEPs were mapped to four ear trait- and four kernel trait-related QTL, respectively. (Table 3, Supplementary Tables S8 and S9). Further investigation revealed that 265 (59.7%) of the mapped DEPs belonged to the parental expression-level dominance category, with 80 (18.3%) HD568 dominance proteins and 185 (42.4%) Z58 dominance proteins (Supplementary Tables S8 and S9).

**Table 3:** DEPs mapped to ear trait-related QTL.

Traits^a^	Density^b^	QTL^c^	Marker interval	LOD^d^	PVE (%)^e^	DEPs
EL	LPD	*qLel1*	SNP0036–SNP0038	2.93	4.37	**6**
	LPD	*qLel2*	SNP0405–SNP0404	6.54	10.07	**0**
	LPD	*qLel3*	SNP0741–SNP0738	6.24	10.50	**8**
	MPD	*qMel3*	SNP0880–SNP0877	3.35	6.17	**15**
	HPD	☆*qHel2*	SNP0408–SNP0406	3.48	5.80	**4**
	HPD	*qPHel3*	SNP0745–SNP0748	2.70	4.47	**0**
	HPD	*qHel4*	SNP0975–SNP0969	5.75	9.77	**3**
	HPD	*qHel9	SNP2707–SNP2712	2.65	4.30	**14**
ED	LPD/MPD	*qLed1*/*qMed1*	SNP0122–SNP0128	3.45	5.26/5.34	**6**
	LPD	*qLed2*	SNP0397–SNP0396	3.22	4.74	**6**
	LPD	*qLed3-1*	SNP0891–SNP0887	3.05	5.28	**14**
	LPD	*qLed3-2*	SNP0915–SNP0911	5.81	8.88	**11**
	LPD	*qLed4*	SNP1363–SNP1359	4.39	7.16	**4**
	MPD	*qMed2*	SNP0605–SNP0609	5.60	8.62	**2**
	MPD	*qMed3*	SNP0865–SNP0857	3.50	5.72	**18**
	MPD	△*qMed4*	SNP1302–SNP1322	5.60	8.53	**3**
	MPD	*qMed5*	SNP1606–SNP1610	2.85	4.20	**0**
	HPD	*qHed1*	SNP0015–SNP0019	3.98	6.91	**54**
	HPD	*qHed2*	SNP0388–SNP0387	3.23	5.58	**1**
	HPD	*qHed5*	SNP1395–SNP1399	3.93	8.69	**19**
	HPD	*qHed9	SNP2707–SNP2712	5.00	9.05	**14**
ERN	LPD/MPD/HPD	*qLern2*/*qMern2*/*qHern2*	SNP0480–SNP0490	4.69	7.56/8.84/8.79	**4**
	LPD	*qLern3-1*	SNP0849–SNP0850	2.73	4.29	**3**
	LPD/MPD	*qLern3-2*/*qMern3-2*	SNP0916–SNP0915	4.26	7.32/5.58	**3**
	LPD/MPD	*qLern4*/*qMern4*	SNP1316–SNP1287	6.54	10.88/14.07	**7**
	MPD	*qMern3-1*	SNP0851–SNP0848	3.37	5.25	**11**
	MPD	*qMern7*	SNP2004–SNP2009	2.95	4.34	**11**
	HPD	*qHern10*	SNP3060–SNP3055	3.30	5.99	**15**
	HPD	*qHern3*	SNP0737–SNP0736	5.72	8.43	**1**
	HPD	△*qHern4*	SNP1302–SNP1322	5.07	7.67	**3**
KNPR	LPD	*qLknpr1*	SNP0065–SNP0066	2.74	4.78	**1**
	LPD	☆*qLknpr2*	SNP0408–SNP0406	2.73	4.85	**4**
	LPD	*qLknpr6*	SNP1959–SNP1954	4.86	8.63	**8**
	MPD	*qMknpr5*	SNP1393–SNP1395	2.58	5.50	**10**
	MPD/HPD	*qMknpr7*/*qHknpr7*	SNP2211–SNP2203	3.12	6.25/8.06	**13**

^a^EL, ear length; ED, ear diameter; ERN, ear row number; KNPR, Kernel number per row; ^b^LPD, low planting density; MPD, Medium planting density; HPD, high planting density. ^c^QTL, q + planting density abbreviation + trait abbreviation + chromosome number + QTL number, e.g., qLed3-1,corresponds to the first QTL for EL on chromosome 3; ^d^Logarithm of odds for each QTL; ^e^PVE, phenotypic variation explained; *, Δ, ☆QTL associated with different traits located within the same confidence marker intervals.

Detailed inspection of DEPs located into the QTL intervals has revealed some interesting proteins that may play importance role in ear or kernel development. For ear traits, five overdominance proteins (e.g., glycosyltransferase, asparaginase and glutamate synthase) and four HPD proteins (e.g., D-3-phosphoglycerate dehydrogenase and phosphofructokinase), which mainly participated in amino acid and nitrogen metabolism were mapped into ear diameter-related QTL (*qLed2*, *qLed3-1* and *qMed3*) and ear length-related QTL (*qMel3*) intervals, respectively. Active expression of these proteins in hybrid would highly increase the nitrogen use efficiency in maize ear and promote the ear growth rate. In addition, seven kinase, (e.g., leucine-rich repeat receptor-like protein kinase, serine/threonine-protein kinase), and five transcript factors (e.g., bZIP, MYB, MYC), which expressed at overdominance or high parent dominance level in hybrid, were also found located in ear diameter-related QTL intervals (Supplementary Tables S8). These proteins may play importance role in hormone response and cell signaling and are required for enhancing cell size and the rate of ear development^55^. For kernel traits, two cell cycle-related proteins, which expressed at high parent dominance pattern, were found to be mapped into kernel length QTL (*qHkl9*) interval (Supplementary Tables S9). The rate and duration of cell division cycles have been reported to play an important role in maize kernel size^56^, keep the high expression level of beneficial allele in hybrid may lead to superior performance in kernel length. These results support our DEP identification and suggest potential functions in ear trait heterosis. The large number of mapped DEPs showed parental expression-level dominance and confirmed that ELD proteins may play more important roles in hybrid vigor.

## Discussion

As an important biological phenomenon, heterosis has been extensively studied from genomic, transcriptomic, and epigenetic perspectives^2,30,57^. A comprehensive proteomics profiling of the end products of transcription should be useful in elucidating the biological mechanisms underlying heterosis. In this study, we conducted genome-wide profiling of the protein expression divergence between the maize hybrid ZD909 and its parents. Immature ears at V14 stage were chosen because strong heterosis has been discovered in ZD909 ear architecture traits (Fig. 1 and Table 1), which are highly correlated with maize yield.

Our study detected 9,713 proteins expressed in all three genotypes; 4,135 (42.6%) proteins showed differential expression between hybrid ZD909 and its parents, providing evidence of high expression divergence between the hybrid and its parent lines (Table 2). In previous 2-DE gel studies, only a few hundred to two thousand proteins were identified in root, seed, leaf, and ear shoot tissues of rice, wheat, and maize. Among them, 10% to 36.9% of proteins were differentially accumulated between the hybrid and its parent lines^33–35,58^. Our study using TMT label-based approaches provided more precision and greater identification of differentially expressed proteins. Among these differentially expressed proteins, 3,183, 2,926, and 2,011 DEPs were found between HD568 and Z58, ZD909 and HD568, and ZD909 and Z58, respectively (Table 2). Higher number of DEPs between the two parent lines suggested great genetic divergence between HD568 and Z58, which could provide sufficient allele diversity for hybrid vigor. More DEPs were identified between ZD909 and HD568 compared to that between ZD909 and Z58, which suggested that protein expression in ZD909 is more similar to the maternal line Z58. The hierarchical cluster analysis further confirms the assumption (Fig. 2). These expression results, together with the vigorous ear phenotype of Z58, imply that Z58 alleles may contribute greatly to ZD909 ear heterosis.

In previous studies, multiple gene or protein expression patterns, including additive, high- and low-parent dominance, overdominance, and underdominance, have been implicated in heterosis manifestation^17,18,24^. To address the correlation between differential gene expression patterns and heterosis, genome-wide transcriptome studies have been widely conducted in various tissues of maize, rice, wheat and other crops. Many studies have reported that additively expressed genes were more prevalent and positively associated with hybrid yield and heterosis^17,24,59^. However, in other studies, nonadditive gene expression patterns were considered important in conferring novel or superior hybrid performance^27,28^. From a proteomic perspective, most previous studies were primarily interested in nonadditive expression patterns that may coordinately produce a heterotic phenotype. In 3.5 d primary maize roots, 49% of the most abundant soluble proteins accumulated differently from additivity in the hybrid, and 51% of the nonadditively accumulated proteins displayed above high-parent or below low-parent expression^32^. In dry and 24 h imbibed seed embryos of maize, 47.01% and 34.55% protein spots displayed nonadditively expressed patterns, respectively^33^. Another study of germinating seeds after 24 h of soaking for five elite maize hybrids and their parents revealed that 80.06%, 58.74%, 79.95%, 54.50%, and 46.30% of nonadditively expressed proteins were identified in the hybrids Zhengdan 958, Nongda108, Yuyu 22, Xundan 20, and Xundan 18, respectively^60^. A time course investigation of leaf proteomics at tillering, flowering and grain-filling stages of rice discovered that 36.6–46.2% of differentially expressed genes displayed nonadditive expression patterns^34^. These results indicated that the proportions of gene expression patterns varied among species, tissues, and developmental stages and were also affected by different experimental designs and statistical methods. There are no universal rules fully governing the correlation between differential gene expression patterns and heterosis for every phenomenon or organism. Till now, a comprehensive proteomic study of relationship between differential protein expression patterns and maize ear development heterosis is still lacking.

In this study, 2,698 of 3,258 (82.8%) DEPs were identified as nonadditively expressed in immature ears of hybrid ZD909 and 76.8% (2,073) of nonadditively expressed proteins were expressed at ELD pattern (Fig. 3 and Supplementary Table S2). Among the ELD proteins, 1,080 and 993 proteins were classified as high parent dominance and low parent dominance expression, respectively. The proportions of the patterns underscored the importance of dominance expression and suggested that ZD909 may preserve the high expression levels of favorable alleles from the two parents, which contribute greatly to the superior phenotypes. In transcriptome studies, allelic expression bias was observed for 50% to 60% of genes assayed in maize hybrids^61,62^ and was suggested to be a source of gene expression divergence and phenotypic novelty. KEGG pathway enrichment analysis results revealed that ‘metabolic pathways’, ‘biosynthesis of secondary metabolites’, ‘carbon metabolism’, ‘glycolysis/gluconeogenesis’, and ‘biosynthesis of amino acids’ were significantly overrepresented in high parent dominance proteins, while no pathway was enriched in the low parent expression proteins. (Fig. 4b and Supplementary Table S4).

At V14 stage, the hybrid ZD909 exhibit larger ear size compared to its parents, implying high rate of cell growth and dry matter accumulation, which highly depend on the active energy metabolism. The vigorous ear of ZD909 also serve as a temporary storage organ and conveyor of carbohydrates and nitrogen nutrients for florets and kernels during the reproductive stage^63,64^. Thus efficient carbohydrate metabolism and nitrogen assimilation are essential for maize ear development and subsequent kernel set and filling and ultimately determine the maize grain yield^65^. In our KEGG results, carbon metabolism, glycolysis and pentose phosphate are enzymatic pathways essential to carbohydrate metabolism, and directly affecting ear growth, dry matter accumulation and grain yield. Biosynthesis of amino acids are important for carbohydrate portioning in the ear; the amino acid interchange and metabolism in the ear at the V14 stage play crucial roles in determining sink strength of the ear.

Detailed examination of the proteins in the ‘metabolic pathways’ from the HPD category also found that many DEPs were involved in carbohydrate and energy metabolism, amino acid and nitrogen metabolism, and lipid metabolism (Supplementary Table S5). For example, fructose-bisphosphate aldolase, fructokinase, hexokinase, glyceraldehyde-3-phosphate dehydrogenase, malate dehydrogenase, 6-phosphogluconate dehydrogenase, and isocitrate dehydrogenase are key enzymes involved in the glycolysis, citrate or tricarboxylic acid cycle and oxidative pentose phosphate pathways (Supplementary Table S5). These proteins play crucial roles in carbohydrate portioning in maize ears. Glutamine synthetase, glutamate dehydrogenase, aminotransferase and aspartate kinase are important for amino acid and nitrogen metabolism (Supplementary Table S5). Glutamine synthase has been reported to control the nitrogen use efficiency (NUE) with respect to both ear development and kernel filling^63,65^. Asparagine synthetase is of major importance for C and N translocation between the maize ear and the developing kernels^66,67^. In addition, lipid metabolism-related proteins such as long-chain-fatty-acid-CoA ligase, diacylglycerol kinase and phospholipase D were also expressed in high parent dominance pattern (Supplementary Table S5). At the V14 stage, a variety of abiotic stresses may negatively affect ear development, resulting in lower fertilization and decreased final grain yield. Phospholipase D and diacylglycerol kinase are responsible for the formation of the signaling lipid phosphatidic acid (PA), which can respond to environmental cues, activate downstream signaling pathways and lead to stress resistance^68^. Based on the above, we conclude that hybrid ZD909 tends to maintain the high expression level of superior alleles, which mainly function in carbohydrate metabolism, nitrogen assimilation, and lipid metabolism that contribute greatly to maize ear development and hybrid vigor at this developmental stage.

To verify the validation of our proteomic results and investigate the links between DEPs, QTL and heterosis, we mapped 3,752 DEPs between ZD909 and its parents to the QTL region and found that 265 and 179 DEPs corresponded to four ear trait- and four kernel trait-related QTL, respectively (Table 3, Supplementary Tables S8 and S9). In addition, 265 (59.7%) of the mapped DEPs belonged to the parental expression-level dominance category. Our results suggested that these DEPs have a potential role in regulating ear and kernel traits. The large proportion of mapped DEPs showed parental expression-level dominance further proved that ELD proteins may play more important roles in hybrid vigor. Several interesting DEPs involved in amino acid and nitrogen metabolism, such as glycosyltransferase, asparaginase, glutamate synthase and phosphofructokinase, were mapped into ear diameter and ear length-related QTL intervals (Supplementary Tables S8 and S9). These results also help to narrow the QTL region to a few genes and provide useful information for subsequent gene cloning.

In conclusion, our study suggested a possible mechanism for heterosis of maize immature ear development. Large genetic divergence between two parents have provide abundant source of superior alleles. Hybrid ZD909 tend to preserve the dominance expression pattern of the favorable alleles, which mainly participated in carbohydrate metabolism and nitrogen assimilation, and express more vigorous performance relative to its parents. The active C and N metabolism in hybrid could provide sufficient energy and nutritious for the rapid growth and dry matter accumulation in ZD909 ear, giving rise to a large “sink” for subsequent kernel set and grain yield formation.

## Methods

### Plant Materials

Maize hybrid ZD909 and its parental lines Z58 and HD568 were grown in the spring of 2014 at Changping Experimental Station in Beijing (40°13′N 116°15′E). The field planting followed a randomized complete block design (RCBD) with three replicates per genotype. Each plot consisted of 5 rows, 0.6 m apart and 3 m long. A total of 12 plants per genotype were grown at a row, with a plant density of 67,500 plants per hectare. The soil moisture was maintained at greater than 70% of the field water capacity by irrigation. Nitrogen, phosphorus and potassium were applied at 200 kg N ha^−1^, 60 kg P2O5 ha^−1^ and 90 kg K2O ha^−1^ as basal fertilizers. Three upmost ears at 14-leaf stage (V14) were harvested for each genotype per biological replicate and observed with microscope. The samples were collected along the path of the diagonal of each plot while avoiding the first and last rows. At V14 stage, the immature ears are undergoing floral organs differentiation with an increase in ear size and the rapid elongation of pistils. To make sure the tissues we sampled at same development stage, only ears whose silks reached one third of the ear length were selected. After manual removal of husks and silks, ears were frozen in liquid nitrogen immediately.

### Protein Preparation

One gram of fresh maize ears were ground into fine power in liquid nitrogen. The grounded tissues were suspended in ice-cold acetone containing 10% (w/v) trichloroacetic acid for 16 h at −20 °C, then centrifuged at 16,000 × g for 15 min. The pellets were resuspended in ice-cold acetone, and incubated for 2 h at −20 °C, then centrifuged at 16,000 × g for 15 min at 4 °C. This step was repeated for three times. Then, the pellets were lyophilized. The crude protein powders were solubilized in lysis buffer (8 M urea, 0.1%SDS, 1 mM PMSF and 1 × PI) for 1 h at 4 °C, then centrifuged at 16,000 × g for 10 minutes at 4 °C. The supernatants were transferred into a new tube with the solution replacement by ultrafiltration, and the protein concentration was determined using bicinchoninic acid (BCA) assay.

### Tandem Mass Tag (TMT) labeling

Tandem mass tags TMT10 (Pierce, USA) with various reporter ions (126–131 Da) were applied as isobaric tags for relative quantification, and TMT labeling was performed according to the manufacturer’s instructions. Briefly, 100 μg per condition was transferred into a new tube, and 100 mM triethyl ammonium bicarbonate (TEAB) buffer was added to the protein solution to a final volume of 100 μL. Then, 5 μL of 200 mM tris (2-carboxyethyl)phosphine (TCEP) was added and the sample was incubated at 55 °C for 1 hour followed by the addition of 5 μL of 375 mM iodoacetamide to the sample and incubation for 30 min protected from light at room temperature. Proteins were precipitated with pre-chilled (−20 °C) acetone. After resuspension with 100 μL of 100 mM TEAB, proteins were digested overnight at 37 °C with 2.5 μg trypsin (Sigma, USA). The digested samples were individually labeled with TMT10 reagents at room temperature for 1 h. The labeling reaction was quenched by adding 8 μL of 5% hydroxylamine. Finally, labeled peptide aliquots were combined for subsequent fractionation.

### Fractionation of labeled peptides

The labeled samples were lyophilized and dissolved in solvent A (2% acetonitrile and 98% H_2_O, pH 10), loaded onto an Xbridge PST C18 Column (130 Å, 5 μm, 250 × 4.6 mm column, Waters, USA) and separated using a linear gradient of 0–95% solvent B (90% acetonitrile and 10% H_2_O, pH 10) at a flow rate of 1 mL/min for 67 min. A total of 20 components were obtained by merging samples from 24 min to 63 min according to the chromatogram map. The samples were concentrated by vacuum centrifugation and stored at −80 °C until LC-MS/MS analysis.

### LC-MS/MS analysis

The liquid chromatography-mass spectrometry (LC-MS/MS) analysis was carried out with a Q Exactive mass spectrometer (Thermo Scientific, USA) based on CapitalBio Technology. A DIONEX nano-UPLC system with an Acclaim C18 PepMap100 nano-Trap column (75 μm × 2 cm, 2 μm particle size, Thermo Scientific, USA) connected to an Acclaim PepMap RSLC C18 analytical column (75 μm × 25 cm, 2 μm particle size) (Thermo Scientific, USA) was used for the separation of the peptide mixture by reverse phase chromatography. The sample was first dissolved in solution containing 4% acetonitrile and 0.1% formic acid and then loaded on the column. The linear gradient of mobile phase B (0.1% formic acid in 99.9% acetonitrile) was run at a flow rate of 300 μl/min. The LC gradient was initiated with a ramp of 3 to 30% B in 43 min and followed by a steep increase to 80% B in 1 min. The Q Exactive mass spectrometer was performed in data-dependent mode. All mass spectra were acquired with full scans (350–1,600 m/z) using an Orbitrap mass analyzer at a mass resolution of 70,000 at 400 m/z. The twenty most intense precursor ions detected in the full MS survey scan were selected for higher energy collision-induced dissociation (HCD) and analyzed in the Orbitrap at resolution of 35,000 at an m/z of 400. Dynamic exclusion was set for 18 s.

### Protein identification and quantification

The raw mass data were processed and searched against the NCBI’s RefSeq protein sequence database of *Zea mays* (taxid: 4577) using Proteome Discoverer software (version 1.4). Searches were performed using the following criteria: Trypsin was designated as the enzyme and the missing of two cleavage sites was allowed. The precursor mass tolerance was set to 15 ppm, and fragment ion mass tolerance was set to 20 mmu. Carbamidomethylation of cysteine residues and TMT modification of N-terminus and lysine residues were selected as fixed modifications; methionine oxidation was selected as variable modifications. The score threshold for peptide identification was set at 0.01 false-discovery rate (FDR) based on a target-decoy approach. Reporter ions were quantified with a mass tolerance window of 20 ppm using the most confident centroid. For protein quantification, only unique peptides were taken into consideration.

### Identification and classification of differential protein expression patterns

One-way ANOVA (FDR-adjusted p-value of 0.05) with a Bonferroni post hoc test was performed to detect significantly differentially expressed proteins (DPEs) among ZD909, HD568 and Z58. Classification of the protein expression patterns revealed four main categories: additive, parental expression-level dominance, overdominance, and underdominance. Proteins in hybrids with expression values between the two parent lines were considered to indicate additive expression. Proteins with expression values not significantly different from one parent but significantly higher (or lower) than the other parent, were considered to exhibit expression-level dominance. Proteins with significantly higher (or lower) expression than both inbred line parents were considered to exhibit overdominance (underdominance). The detailed gene actions were further classified into 12 classes and presented in 2 dimensions. The normalized protein abundance was used to calculate fold changes. The radius represents fold changes between the highest and lowest expression levels among the three genotypes.

### Kyoto Encyclopedia of Genes and Genomes (KEGG) pathway enrichment analysis

Kyoto Encyclopedia of Genes and Genomes (KEGG) pathway enrichment analysis was conducted using KOBAS 3.0 (http://kobas.cbi.pku.edu.cn/). The KEGG pathways with a Benjamini and Yekutieli corrected p-value lower than 0.01 were considered as significant.

### Data Availability

All data generated or analyzed during this study are included in this published article and its Supplementary Information files.

## Electronic supplementary material

Supplementary Information

Supplementary table 1
