# Supplementary material for: Genome-wide proteomic profiling reveals the role of dominance protein expression in heterosis in immature maize ears
Source: Sci Rep. 2017 Nov 23;7:16130. doi: 10.1038/s41598-017-15985-3 (PMC5700959; doi:10.1038/s41598-017-15985-3)
Supplement: Supplementary file 1 — Supplementary Information [file 41598_2017_15985_MOESM1_ESM.pdf]

Supplementary Information

Genome-wide proteomic profiling reveals the role of dominance protein expression in heterosis in immature maize ears

Xiaojiao Hu[#], Hongwu Wang[#], Kun Li, Yujin Wu, Zhifang Liu[*]& Changling Huang[*]

Institute of Crop Science, Chinese Academy of Agricultural Sciences, National Engineering Laboratory for Crop Molecular Breeding, Beijing, 100081, China

[#]Theses authors contributed equally to this work.

*To whom correspondence should be addressed to:

Changling Huang

Tel.: +86 (0)10 82108738

Email: huangchangling@caas.cn

Or Zhifang Liu

Tel.: +86 (0)1082108629

Email: liuzhifang@caas.cn

Address: Institute of Crop Science, Chinese Academy of Agricultural Sciences, No.12 Zhongguancun South Main Street, Beijing, 100081, China

Supplementary Table S2 Protein expression patterns in hybrid ZD909

Supplementary Table S3 Well-known genes expressed in immature ears of ZD909 and its parents

Supplementary Table S4 KEGG pathways enriched in nonadditively expressed proteins

Supplementary Table S5 Differentially expressed proteins under each pathway enriched in high parent dominance category

Supplementary Table S6 Differentially expressed proteins under each pathway in overdominance category

Supplementary Table S7 Differentially expressed proteins under each pathway in underdominance category

Supplementary Table S8 DEPs mapped to ear trait-related QTLs

Supplementary Table S9 DEPs mapped to kernel trait-related QTLs

Supplementary Table S2 Protein expression patterns in hybrid ZD909

				**Criteria**			
**Pattern**		**Class**	**Clock**	**HD568≠Z58**	**ZD909≠HD568**	**ZD909≠Z58**	**Number**
**conserved**		Z58<ZD909<HD568	3	Y	Y	Y	266
		HD568<ZD909<Z58	9	Y	Y	Y	294
**parental expression level domiance**	HP-dominance-HD568	Z58<HD568=ZD909	2	Y	N	Y	333
	HP-dominance-Z58	HD568<ZD909=Z58	10	Y	Y	N	747
	LP-dominance-HD568	HD568=ZD909<Z58	8	Y	N	Y	332
	LP-dominance-Z58	Z58=ZD909<HD568	4	Y	Y	N	661
**over-dominance**		Z58<HD568<ZD909	1	Y	Y	Y	17
		HD568<Z58<ZD909	11	Y	Y	Y	58
		HD568=Z58<ZD909	12	N	Y	Y	247
**under-dominance**		ZD909<Z58<HD568	5	Y	Y	Y	65
		ZD909<HD568=Z58	6	N	Y	Y	210
		ZD909<HD568<Z58	7	Y	Y	Y	28
**ambiguous**		HD568=Z58, HD568=ZD909, ZD909<Z58	None	N	N	Y	81
		HD568=Z58, HD568=ZD909, Z58<ZD909	None	N	N	Y	80
		HD568<Z58, HD568=ZD909, ZD909=Z58	None	Y	N	N	195
		Z58<HD568, HD568=ZD909, ZD909=Z58	None	Y	N	N	188
		HD568=Z58, Z58=ZD909, ZD909<HD568	None	N	Y	N	166
		HD568=Z58, Z58=ZD909, HD568<ZD909	None	N	Y	N	168
Total							4136

The classes 1–12 have been designed to evaluate the expression levels in the hybrids; (Y) yes, (N) no.

Supplementary Table S3 Well-known genes expressed in immature ears of ZD909 and its parents

GI	Genes	Full Name	Differential expression patterns
226507794	*IFA1*	Indeterminate floral apex1	HP-dominance-HD568
673921565	*MADS15*	MADS-box transcription factor 14	HP-dominance-HD568
162460316	*ZAG1*	Zea AGAMOUS1	Overdominance
162458955	*TASSELLESS1*	Tasselless1	Overdominance
806904685	*ZAG2*	Zea AGAMOUS homolog2	Overdominance
818213441	*TSH4*	Tasselsheath4	Underdominance
162458085	*KNOTTED-1*	Homeotic protein knotted-1	Underdominance
162459099	*ZFL1*	Zea floricaula/leafy1	LP-dominance-Z58
874507460	*D8*	DELLA protein DWARF8	None
670440195	*MADS8*	MADS-box transcription factor 8	None
670407211	*TD1*	Thick tassel dwarf1	None
670396163	*TGA1*	Teosinte glume architecture1	None
304555573	*REL2*	Ramosa 1 enhancer locus 2	None
226506516	*RA1*	Ramosa1	None
162463623	*MADS29*	MADS-box transcription factor 29	None
162462916	*FASCIATED EAR2*	Fasciated ear2	None
162461674	*ZAG3/BDE1*	Zea AGAMOUS3; bearded-ear1	None
162457897	*MADS15*	MADS-box transcription factor 15	None

Supplementary Table S4 KEGG pathways enriched in nonadditively expressed proteins

Term	ID	Percentage	Corrected P-Value
**HD568 dominance**			
Metabolic pathways	zma01100	13.5%	1.53E-13
Biosynthesis of secondary metabolites	zma01110	7.5%	1.09E-06
Carbon metabolism	zma01200	3.3%	2.91E-06
Biosynthesis of amino acids	zma01230	2.7%	0.000623
Glycolysis / Gluconeogenesis	zma00010	1.8%	0.001947
Pyruvate metabolism	zma00620	1.4%	0.00655
Linoleic acid metabolism	zma00591	0.6%	0.008664
**Z58 dominance**			
Metabolic pathways	zma01100	11.9%	2.78E-21
Biosynthesis of secondary metabolites	zma01110	6.7%	2.82E-11
Biosynthesis of amino acids	zma01230	2.7%	5.41E-09
Carbon metabolism	zma01200	2.6%	1.58E-08
RNA transport	zma03013	1.7%	3.84E-05
Pyrimidine metabolism	zma00240	1.3%	0.000428
Carbon fixation in photosynthetic organisms	zma00710	1.0%	0.000556
Pentose phosphate pathway	zma00030	0.9%	0.000556
Amino sugar and nucleotide sugar metabolism	zma00520	1.2%	0.001974
Glycolysis / Gluconeogenesis	zma00010	1.2%	0.002727
DNA replication	zma03030	0.9%	0.003251
Purine metabolism	zma00230	1.3%	0.004846
2-Oxocarboxylic acid metabolism	zma01210	0.7%	0.009861
alpha-Linolenic acid metabolism	zma00592	0.6%	0.009861
Spliceosome	zma03040	1.3%	0.009861
Valine, leucine and isoleucine biosynthesis	zma00290	0.4%	0.009861
**High parent dominance**			
Metabolic pathways	zma01100	15.3%	2.49E-06
Linoleic acid metabolism	zma00591	0.7%	5.10E-06
Biosynthesis of secondary metabolites	zma01110	9.6%	1.75E-05
Carbon metabolism	zma01200	3.4%	3.89E-05
Biosynthesis of antibiotics	zma01130	5.0%	8.38E-05
Amino sugar and nucleotide sugar metabolism	zma00520	2.0%	1.84E-04
Glycolysis / Gluconeogenesis	zma00010	2.0%	1.99E-04
Biosynthesis of amino acids	zma01230	3.3%	2.09E-04
Pentose phosphate pathway	zma00030	1.1%	9.99E-04
**Overdominance**			
Metabolic pathways	zma01100	11.2%	0.005402
Biosynthesis of secondary metabolites	zma01110	7.5%	0.00693
**Underdominance**			
Ribosome biogenesis in eukaryotes	zma03008	2.6%	0.000288

Supplementary Table S5 Differentially expressed proteins under each pathway in high parent dominance category

Pathways	Protein ID	Annotation	Expression pattern
**metabolic pathways**	**photosynthesis and the calvin cycle**		
	11467204	cytochrome f (chloroplast)	HP-dominance-Z58
	162463469	ribulose bisphosphate carboxylase small chain, chloroplastic precursor	HP-dominance-Z58
	226529672	triosephosphate isomerase, cytosolic	HP-dominance-Z58
	226532904	ribulose bisphosphate carboxylase small subunit 2	HP-dominance-Z58
	670429453	fructose-bisphosphate aldolase 3, chloroplastic	HP-dominance-HD568
	890782319	fructose-bisphosphate aldolase cytoplasmic isozyme	HP-dominance-HD568
	**glycolysis and gluconeogenesis**		
	162457852	pyruvate decarboxylase 3	HP-dominance-HD568
	162458262	dihydrolipoamide S-acetyltransferase	HP-dominance-HD568
	162459678	phosphoglucomutase, cytoplasmic 2	HP-dominance-Z58
	162460546	alcohol dehydrogenase 2	HP-dominance-Z58
	212274473	cinnamyl alcohol dehydrogenase 1	HP-dominance-HD568
	212721344	putative cinnamyl-alcohol dehydrogenase family protein	HP-dominance-Z58
	226494977	pyruvate dehydrogenase E1 component alpha subunit	HP-dominance-Z58
	226495363	apospory-associated protein C	HP-dominance-HD568
	226496759	pyruvate kinase, cytosolic isozyme	HP-dominance-Z58
	226498808	uncharacterized protein LOC100279587	HP-dominance-HD568
	226502865	uncharacterized protein LOC100282820	HP-dominance-HD568
	226528689	uncharacterized protein LOC100273217	HP-dominance-Z58
	226529672	triosephosphate isomerase, cytosolic	HP-dominance-Z58
	226530882	glucose-6-phosphate isomerase	HP-dominance-Z58
	226532381	6-phosphofructokinase	HP-dominance-Z58

293331181	uncharacterized protein LOC100383094 precursor	HP-dominance-HD568
293333684	uncharacterized protein LOC100382805	HP-dominance-Z58
670388124	hexokinase-3	HP-dominance-HD568
670389993	phosphoglucomutase, chloroplastic	HP-dominance-Z58
670404004	short chain alcohol dehydrogenase 1 isoform X1	HP-dominance-Z58
670429115	2,3-bisphosphoglycerate-independent phosphoglycerate mutase isoform X1	HP-dominance-HD568
670429453	fructose-bisphosphate aldolase 3, chloroplastic	HP-dominance-HD568
670432709	hexokinase-3	HP-dominance-Z58
670436652	enolase isoform X1	HP-dominance-Z58
890782319	fructose-bisphosphate aldolase cytoplasmic isozyme	HP-dominance-HD568
**citrate or tricarboxylic acid cycle**		
162462055	malate dehydrogenase [NADP], chloroplastic precursor	HP-dominance-HD568
212275278	uncharacterized protein LOC100191841	HP-dominance-Z58
226493349	3-isopropylmalate dehydratase large subunit 2	HP-dominance-Z58
226494977	pyruvate dehydrogenase E1 component alpha subunit	HP-dominance-Z58
226499456	uncharacterized LOC100280203	HP-dominance-Z58
226502058	malate dehydrogenase 2	HP-dominance-HD568
226506808	uncharacterized protein LOC100279871	HP-dominance-HD568
226533417	uncharacterized protein LOC100286036	HP-dominance-Z58
**amino acid and nitrogen metabolism**		
162462912	4-hydroxy-tetrahydrodipicolinate synthase, chloroplastic precursor	HP-dominance-Z58
219363693	cysteine synthase	HP-dominance-HD568
226493349	3-isopropylmalate dehydratase large subunit 2	HP-dominance-Z58
226493858	dihydrodipicolinate synthase 2	HP-dominance-Z58
226495187	uncharacterized protein LOC100284589	HP-dominance-HD568

226496759	pyruvate kinase, cytosolic isozyme	HP-dominance-Z58
226497150	ribulose-phosphate 3-epimerase	HP-dominance-HD568
226499456	ATP binding / ATP citrate synthase	HP-dominance-Z58
226499532	arogenate dehydratase	HP-dominance-Z58
226499866	L-allo-threonine aldolase	HP-dominance-HD568
226500284	chorismate synthase 2	HP-dominance-Z58
226500444	uncharacterized protein LOC100281708	HP-dominance-Z58
226502522	3-dehydroquinate synthase	HP-dominance-Z58
226502865	uncharacterized protein LOC100282820	HP-dominance-HD568
226504284	glutamyl-tRNA(Gln) amidotransferase subunit A, chloroplastic/mitochondrial	HP-dominance-HD568
226508518	3-isopropylmalate dehydratase small subunit 2	HP-dominance-Z58
226529672	triosephosphate isomerase, cytosolic	HP-dominance-Z58
226529888	2-isopropylmalate synthase B	HP-dominance-Z58
226531522	uncharacterized protein LOC100286153	HP-dominance-Z58
226532249	phospho-2-dehydro-3-deoxyheptonate aldolase 1	HP-dominance-Z58
226532381	6-phosphofructokinase	HP-dominance-Z58
293335401	uncharacterized protein LOC100381786	HP-dominance-HD568
363543397	glutamine synthetase 1	HP-dominance-HD568
363543401	glutamate dehydrogenase	HP-dominance-HD568
363543511	tryptophan synthase alpha	HP-dominance-Z58
670363597	PREDICTED: probable low-specificity L-threonine aldolase 2 isoform X3	HP-dominance-Z58
670374829	D-3-phosphoglycerate dehydrogenase 3, chloroplastic	HP-dominance-Z58
670377358	putative aconitate hydratase, cytoplasmic isoform X2	HP-dominance-HD568
670429115	2,3-bisphosphoglycerate-independent phosphoglycerate mutase isoform X1	HP-dominance-HD568
670429453	fructose-bisphosphate aldolase 3, chloroplastic	HP-dominance-HD568

670432428	glutamate synthase 1 [NADH], chloroplastic isoform X1	HP-dominance-Z58
670436652	enolase isoform X1	HP-dominance-Z58
806638836	glutamine synthetase root isozyme 2 isoform 1	HP-dominance-HD568
890782319	fructose-bisphosphate aldolase cytoplasmic isozyme	HP-dominance-HD568
949474735	glutamine synthetase root isozyme 4	HP-dominance-Z58
670393682	aminotransferase y4uB isoform X1	HP-dominance-HD568
**pentose phosphate pathways**		
162459678	phosphoglucomutase, cytoplasmic 2	HP-dominance-Z58
226491622	glucose-6-phosphate 1-dehydrogenase, cytoplasmic isoform	HP-dominance-Z58
226497150	ribulose-phosphate 3-epimerase	HP-dominance-HD568
226530882	glucose-6-phosphate isomerase	HP-dominance-Z58
226532381	6-phosphofructokinase	HP-dominance-Z58
293332949	uncharacterized protein LOC100382228	HP-dominance-Z58
293333684	uncharacterized protein LOC100382805	HP-dominance-Z58
525342897	6-phosphogluconate dehydrogenase 2	HP-dominance-HD568
670389993	phosphoglucomutase, chloroplastic	HP-dominance-Z58
670429453	fructose-bisphosphate aldolase 3, chloroplastic	HP-dominance-HD568
840086659	NADPH producing dehydrogenase of the oxidative pentose phosphate pathway	HP-dominance-Z58
890782319	fructose-bisphosphate aldolase cytoplasmic isozyme	HP-dominance-HD568
**lipid metabolism**		
670385179	phospholipase D alpha 1 isoform X1	HP-dominance-HD568
670405932	phosphatidylinositol 4-kinase alpha 1	HP-dominance-Z58
670420361	phospholipase D family protein isoform X1	HP-dominance-Z58
163838702	uncharacterized protein LOC100127508	HP-dominance-HD568
212722458	Enoyl-[acyl-carrier-protein] reductase [NADH] chloroplastic	HP-dominance-HD568

	226494375	1,2-diacylglycerol 3-beta-galactosyltransferase	HP-dominance-Z58
	226496759	pyruvate kinase, cytosolic isozyme	HP-dominance-Z58
	226500770	long-chain-fatty-acid-CoA ligase	HP-dominance-Z58
	226501716	long-chain-fatty-acid-CoA ligase	HP-dominance-Z58
	226502865	pyruvate kinase	HP-dominance-HD568
	226505666	Neutral/alkaline non-lysosomal ceramidase precursor	HP-dominance-HD568
	226533238	glycosyl transferase, group 1 family protein	HP-dominance-Z58
	226533562	phospholipase D family protein	HP-dominance-Z58
	293333728	uncharacterized protein LOC100383275	HP-dominance-Z58
	670362781	long chain base biosynthesis protein 1a-like	HP-dominance-HD568
	670417395	phospholipase D zeta 1	HP-dominance-Z58
	**jasmonate synthesis -degradation**		
	162460508	allene oxide synthase 1	HP-dominance-Z58
	162464003	lipoxygenase10	HP-dominance-HD568
	162464186	lipoxygenase11	HP-dominance-Z58
	226491696	uncharacterized protein LOC100279433	HP-dominance-Z58
**biosynthesis of secondary metabolites**	890782319	fructose-bisphosphate aldolase cytoplasmic isozyme	HP-dominance-HD568
	840086659	NADPH producing dehydrogenase of the oxidative pentose phosphate pathway	HP-dominance-Z58
	821595499	phenylalanine/tyrosine ammonia-lyase	HP-dominance-Z58
	802084003	glycine dehydrogenase (decarboxylating), mitochondrial precursor	HP-dominance-Z58
	670436652	enolase isoform X1	HP-dominance-Z58
	670436638	uncharacterized protein LOC100502286 isoform X1	HP-dominance-Z58
	670432709	hexokinase-3	HP-dominance-Z58
	670432428	glutamate synthase 1 [NADH], chloroplastic isoform X1	HP-dominance-Z58
	670429453	fructose-bisphosphate aldolase 3, chloroplastic	HP-dominance-HD568

670429115	2,3-bisphosphoglycerate-independent phosphoglycerate mutase isoform X1	HP-dominance-HD568
670420361	phospholipase D family protein isoform X1	HP-dominance-Z58
670417395	phospholipase D zeta 1	HP-dominance-Z58
670404004	short chain alcohol dehydrogenase 1 isoform X1	HP-dominance-Z58
670400213	cycloartenol synthase	HP-dominance-Z58
670397763	probable 4-coumarate--CoA ligase 2	HP-dominance-Z58
670395079	phosphatidate phosphatase PAH2 isoform X2	HP-dominance-HD568
670389993	phosphoglucomutase, chloroplastic	HP-dominance-Z58
670389716	peroxidase 19	HP-dominance-Z58
670388124	hexokinase-3	HP-dominance-HD568
670385179	phospholipase D alpha 1 isoform X1	HP-dominance-HD568
670381038	PREDICTED: uncharacterized protein LOC103648573	HP-dominance-Z58
162460546	alcohol dehydrogenase 2	HP-dominance-Z58
670378841	hydroxymethylglutaryl-CoA synthase	HP-dominance-Z58
670377358	putative aconitate hydratase, cytoplasmic isoform X2	HP-dominance-HD568
670363978	xanthine dehydrogenase	HP-dominance-Z58
670358019	uncharacterized protein LOC100192603 isoform X1	HP-dominance-Z58
661250920	3-hydroxyindolin-2-one monooxygenase	HP-dominance-HD568
525342897	6-phosphogluconate dehydrogenase 2	HP-dominance-HD568
363543511	tryptophan synthase alpha	HP-dominance-Z58
293334775	uncharacterized protein LOC100382972	HP-dominance-Z58
293333684	uncharacterized protein LOC100382805	HP-dominance-Z58
293332949	uncharacterized protein LOC100382228	HP-dominance-Z58
293331181	uncharacterized protein LOC100383094 precursor	HP-dominance-HD568
226533562	phospholipase D family protein	HP-dominance-Z58

226532381	6-phosphofructokinase	HP-dominance-Z58
226532249	phospho-2-dehydro-3-deoxyheptonate aldolase 1	HP-dominance-Z58
226531522	uncharacterized protein LOC100286153	HP-dominance-Z58
226530520	putative cytochrome P450 superfamily protein	HP-dominance-HD568
226529976	Spermidine hydroxycinnamoyl transferase	HP-dominance-HD568
226529888	2-isopropylmalate synthase B	HP-dominance-Z58
226529672	triosephosphate isomerase, cytosolic	HP-dominance-Z58
226508518	3-isopropylmalate dehydratase small subunit 2	HP-dominance-Z58
226507514	uncharacterized protein LOC100282692	HP-dominance-HD568
226506808	uncharacterized protein LOC100279871	HP-dominance-HD568
226504062	fatty acid elongase	HP-dominance-Z58
226502522	3-dehydroquinate synthase	HP-dominance-Z58
226502058	malate dehydrogenase 2	HP-dominance-HD568
226501716	uncharacterized protein LOC100284664	HP-dominance-Z58
226500444	uncharacterized protein LOC100281708	HP-dominance-Z58
226500284	chorismate synthase 2	HP-dominance-Z58
226499866	L-allo-threonine aldolase	HP-dominance-HD568
226499532	uncharacterized protein LOC100281744	HP-dominance-Z58
226499456	uncharacterized LOC100280203	HP-dominance-Z58
226498808	uncharacterized protein LOC100279587	HP-dominance-HD568
226497150	ribulose-phosphate 3-epimerase	HP-dominance-HD568
226496759	pyruvate kinase, cytosolic isozyme	HP-dominance-Z58
226495871	acyltransferase	HP-dominance-Z58
226495363	apospory-associated protein C	HP-dominance-HD568
226495187	uncharacterized protein LOC100284589	HP-dominance-HD568

226494977	pyruvate dehydrogenase E1 component alpha subunit	HP-dominance-Z58
226493858	dihydrodipicolinate synthase 2	HP-dominance-Z58
226493663	peroxidase 39 precursor	HP-dominance-HD568
226493349	3-isopropylmalate dehydratase large subunit 2	HP-dominance-Z58
226493279	inositol monophosphatase 3	HP-dominance-HD568
226493048	uncharacterized protein LOC100283650	HP-dominance-HD568
226491696	uncharacterized protein LOC100279433	HP-dominance-Z58
226491622	glucose-6-phosphate 1-dehydrogenase, cytoplasmic isoform	HP-dominance-Z58
219363693	cysteine synthase	HP-dominance-HD568
219362573	peroxidase 54 precursor	HP-dominance-Z58
212721344	putative cinnamyl-alcohol dehydrogenase family protein	HP-dominance-Z58
212276207	uncharacterized protein LOC100191179	HP-dominance-Z58
212276125	uncharacterized LOC100191549	HP-dominance-Z58
212275836	putative cytochrome P450 superfamily protein	HP-dominance-Z58
212275446	GDP-mannose 35-epimerase	HP-dominance-Z58
212275278	uncharacterized protein LOC100191841	HP-dominance-Z58
212274639	uncharacterized protein LOC100191566 precursor	HP-dominance-Z58
212274473	cinnamyl alcohol dehydrogenase 1	HP-dominance-HD568
212274307	uncharacterized protein LOC100191950 precursor	HP-dominance-Z58
163838702	uncharacterized protein LOC100127508	HP-dominance-HD568
162464362	peroxidase 1	HP-dominance-HD568
162464186	lipoxygenase11	HP-dominance-Z58
162464003	lipoxygenase10	HP-dominance-HD568
162462912	4-hydroxy-tetrahydrodipicolinate synthase, chloroplastic precursor	HP-dominance-Z58
162461460	catalase isozyme 3	HP-dominance-HD568

	162460508	allene oxide synthase 1	HP-dominance-Z58
	162460455	plastid ADP-glucose pyrophosphorylase large subunit	HP-dominance-Z58
	162459763	glossy 8	HP-dominance-Z58
	162459678	phosphoglucomutase, cytoplasmic 2	HP-dominance-Z58
	162458262	dihydrolipoamide S-acetyltransferase	HP-dominance-HD568
	162457852	pyruvate decarboxylase 3	HP-dominance-HD568
**glycolysis and gluconeogenesis**	162457852	pyruvate decarboxylase 3	HP-dominance-HD568
	162458262	dihydrolipoamide S-acetyltransferase	HP-dominance-HD568
	162459678	phosphoglucomutase, cytoplasmic 2	HP-dominance-Z58
	162460546	alcohol dehydrogenase 2	HP-dominance-Z58
	226494977	pyruvate dehydrogenase E1 component alpha subunit	HP-dominance-Z58
	226495363	apospory-associated protein C	HP-dominance-HD568
	226496759	pyruvate kinase, cytosolic isozyme	HP-dominance-Z58
	226498808	uncharacterized protein LOC100279587	HP-dominance-HD568
	226502865	uncharacterized protein LOC100282820	HP-dominance-HD568
	226528689	uncharacterized protein LOC100273217	HP-dominance-Z58
	226529672	triosephosphate isomerase, cytosolic	HP-dominance-Z58
	226530882	glucose-6-phosphate isomerase	HP-dominance-Z58
	226532381	6-phosphofructokinase	HP-dominance-Z58
	293331181	uncharacterized protein LOC100383094 precursor	HP-dominance-HD568
	293333684	uncharacterized protein LOC100382805	HP-dominance-Z58
	670388124	hexokinase-3	HP-dominance-HD568
	670389993	phosphoglucomutase, chloroplastic	HP-dominance-Z58
	670429115	2,3-bisphosphoglycerate-independent phosphoglycerate mutase isoform X1	HP-dominance-HD568
	670429453	fructose-bisphosphate aldolase 3, chloroplastic	HP-dominance-HD568

	670432709	hexokinase-3	HP-dominance-Z58
	670436652	enolase isoform X1	HP-dominance-Z58
	890782319	fructose-bisphosphate aldolase cytoplasmic isozyme	HP-dominance-HD568
**biosynthesis of amino acids**	162462912	4-hydroxy-tetrahydrodipicolinate synthase, chloroplastic precursor	HP-dominance-Z58
	219363693	cysteine synthase	HP-dominance-HD568
	226493349	3-isopropylmalate dehydratase large subunit 2	HP-dominance-Z58
	226493858	dihydrodipicolinate synthase 2	HP-dominance-Z58
	226495187	uncharacterized protein LOC100284589	HP-dominance-HD568
	226496759	pyruvate kinase, cytosolic isozyme	HP-dominance-Z58
	226497150	ribulose-phosphate 3-epimerase	HP-dominance-HD568
	226499456	ATP binding / ATP citrate synthase	HP-dominance-Z58
	226499532	arogenate dehydratase	HP-dominance-Z58
	226499866	L-allo-threonine aldolase	HP-dominance-HD568
	226500284	chorismate synthase 2	HP-dominance-Z58
	226500444	uncharacterized protein LOC100281708	HP-dominance-Z58
	226502522	3-dehydroquinate synthase	HP-dominance-Z58
	226502865	uncharacterized protein LOC100282820	HP-dominance-HD568
	226504284	glutamyl-tRNA(Gln) amidotransferase subunit A, chloroplastic/mitochondrial	HP-dominance-HD568
	226508518	3-isopropylmalate dehydratase small subunit 2	HP-dominance-Z58
	226529672	triosephosphate isomerase, cytosolic	HP-dominance-Z58
	226529888	2-isopropylmalate synthase B	HP-dominance-Z58
	226531522	uncharacterized protein LOC100286153	HP-dominance-Z58
	226532249	phospho-2-dehydro-3-deoxyheptonate aldolase 1	HP-dominance-Z58
	226532381	6-phosphofructokinase	HP-dominance-Z58
	293335401	uncharacterized protein LOC100381786	HP-dominance-HD568
	363543397	glutamine synthetase 1	HP-dominance-HD568

	363543401	glutamate dehydrogenase	HP-dominance-HD568
	363543511	tryptophan synthase alpha	HP-dominance-Z58
	670363597	PREDICTED: probable low-specificity L-threonine aldolase 2 isoform X3	HP-dominance-Z58
	670374829	D-3-phosphoglycerate dehydrogenase 3, chloroplastic	HP-dominance-Z58
	670377358	putative aconitate hydratase, cytoplasmic isoform X2	HP-dominance-HD568
	670429115	2,3-bisphosphoglycerate-independent phosphoglycerate mutase isoform X1	HP-dominance-HD568
	670429453	fructose-bisphosphate aldolase 3, chloroplastic	HP-dominance-HD568
	670432428	glutamate synthase 1 [NADH], chloroplastic isoform X1	HP-dominance-Z58
	670436652	enolase isoform X1	HP-dominance-Z58
	806638836	glutamine synthetase root isozyme 2 isoform 1	HP-dominance-HD568
	890782319	fructose-bisphosphate aldolase cytoplasmic isozyme	HP-dominance-HD568
	949474735	glutamine synthetase root isozyme 4	HP-dominance-Z58
	670393682	aminotransferase y4uB isoform X1	HP-dominance-HD568
**pentose phosphate pathways**	162459678	phosphoglucomutase, cytoplasmic 2	HP-dominance-Z58
	226491622	glucose-6-phosphate 1-dehydrogenase, cytoplasmic isoform	HP-dominance-Z58
	226497150	ribulose-phosphate 3-epimerase	HP-dominance-HD568
	226530882	glucose-6-phosphate isomerase	HP-dominance-Z58
	226532381	6-phosphofructokinase	HP-dominance-Z58
	293332949	uncharacterized protein LOC100382228	HP-dominance-Z58
	293333684	uncharacterized protein LOC100382805	HP-dominance-Z58
	525342897	6-phosphogluconate dehydrogenase 2	HP-dominance-HD568
	670389993	phosphoglucomutase, chloroplastic	HP-dominance-Z58
	670429453	fructose-bisphosphate aldolase 3, chloroplastic	HP-dominance-HD568
	840086659	NADPH producing dehydrogenase of the oxidative pentose phosphate pathway	HP-dominance-Z58
	890782319	fructose-bisphosphate aldolase cytoplasmic isozyme	HP-dominance-HD568
**Carbon metabolism**	890782319	fructose-bisphosphate aldolase cytoplasmic isozyme	HP-dominance-HD568

840086659	NADPH producing dehydrogenase of the oxidative pentose phosphate pathway	HP-dominance-Z58
802084003	glycine dehydrogenase (decarboxylating)	HP-dominance-Z58
670436652	enolase isoform X1	HP-dominance-Z58
670435349	uncharacterized protein LOC100272777 isoform X2	HP-dominance-Z58
670432709	hexokinase-3	HP-dominance-Z58
670429453	fructose-bisphosphate aldolase 3	HP-dominance-HD568
670429115	3-bisphosphoglycerate-independent phosphoglycerate mutase isoform X1	HP-dominance-HD568
670388124	hexokinase-3	HP-dominance-HD568
670377358	putative aconitate hydratase	HP-dominance-HD568
670374829	D-3-phosphoglycerate dehydrogenase 3	HP-dominance-Z58
525342897	6-phosphogluconate dehydrogenase 2	HP-dominance-HD568
363543401	glutamate dehydrogenase	HP-dominance-HD568
293335401	uncharacterized protein LOC100381786	HP-dominance-HD568
293333684	uncharacterized protein LOC100382805	HP-dominance-Z58
293332949	uncharacterized protein LOC100382228	HP-dominance-Z58
226533417	uncharacterized protein LOC100286036	HP-dominance-Z58
226532381	6-phosphofructokinase	HP-dominance-Z58
226530882	glucose-6-phosphate isomerase	HP-dominance-Z58
226529672	triosephosphate isomerase	HP-dominance-Z58
226528689	uncharacterized protein LOC100273217	HP-dominance-Z58
226506808	uncharacterized protein LOC100279871	HP-dominance-HD568
226502865	uncharacterized protein LOC100282820	HP-dominance-HD568
226502058	malate dehydrogenase 2	HP-dominance-HD568
226499456	uncharacterized LOC100280203	HP-dominance-Z58
226498808	uncharacterized protein LOC100279587	HP-dominance-HD568
226497150	ribulose-phosphate 3-epimerase	HP-dominance-HD568

	226496759	pyruvate kinase	HP-dominance-Z58
	226494977	pyruvate dehydrogenase E1 component alpha subunit	HP-dominance-Z58
	226491622	glucose-6-phosphate 1-dehydrogenase	HP-dominance-Z58
	219363693	cysteine synthase	HP-dominance-HD568
	212275278	uncharacterized protein LOC100191841	HP-dominance-Z58
	162463469	ribulose bisphosphate carboxylase small chain	HP-dominance-Z58
	162462055	malate dehydrogenase [NADP]	HP-dominance-HD568
	162461460	catalase isozyme 3	HP-dominance-HD568
	162458262	dihydrolipoamide S-acetyltransferase	HP-dominance-HD568
**Amino sugar and nucleotide sugar metabolism**	162459678	phosphoglucomutase, cytoplasmic 2 [Zea mays]	HP-dominance-Z58
	162460455	plastid ADP-glucose pyrophosphorylase large subunit [Zea mays]	HP-dominance-Z58
	212274887	uncharacterized protein LOC100191391 [Zea mays]	HP-dominance-Z58
	212275097	uncharacterized protein LOC100191463 [Zea mays]	HP-dominance-Z58
	212275438	uncharacterized protein LOC100191846 [Zea mays]	HP-dominance-HD568
	212275446	GDP-mannose 35-epimerase [Zea mays]	HP-dominance-Z58
	212275454	ferric-chelate reductase (NADH)1 [Zea mays]	HP-dominance-Z58
	226493432	uncharacterized protein LOC100282614 [Zea mays]	HP-dominance-Z58
	226498808	uncharacterized protein LOC100279587 [Zea mays]	HP-dominance-HD568
	226503239	RHM1 [Zea mays]	HP-dominance-Z58
	226504710	uncharacterized protein LOC100281192 precursor [Zea mays]	HP-dominance-HD568
	226508708	uncharacterized protein LOC100286359 [Zea mays]	HP-dominance-Z58
	226530882	glucose-6-phosphate isomerase [Zea mays]	HP-dominance-Z58
	293331117	uncharacterized protein LOC100382408 [Zea mays]	HP-dominance-HD568
	293333684	uncharacterized protein LOC100382805 [Zea mays]	HP-dominance-Z58
	670385663	PREDICTED: NADH-cytochrome b5 reductase-like protein [Zea mays]	HP-dominance-Z58
**Biosynthesis of**	890782319	pyruvate decarboxylase 3	HP-dominance-HD568

**antibiotics**	840086659	uncharacterized protein LOC100191463	HP-dominance-Z58
	836939456	bifunctional 3-phosphoadenosine 5-phosphosulfate synthetase 2	HP-dominance-Z58
	802084003	uncharacterized protein LOC100272584	HP-dominance-Z58
	670436652	uncharacterized protein LOC100281744	HP-dominance-Z58
	670432709	uncharacterized protein LOC100282820	HP-dominance-Z58
	670432428	glucose-6-phosphate isomerase	HP-dominance-Z58
	670429453	uncharacterized protein LOC100382805	HP-dominance-HD568
	670429115	putative aconitate hydratase, cytoplasmic isoform X2	HP-dominance-HD568
	670389993	fructose-bisphosphate aldolase 3, chloroplastic	HP-dominance-Z58
	670388124	glucosamine fructose-6-phosphate aminotraansferase 1	HP-dominance-HD568
	670378841	dihydrolipoamide S-acetyltransferase	HP-dominance-Z58
	670377358	uncharacterized protein LOC100191841	HP-dominance-HD568
	670374829	uncharacterized protein LOC100283650	HP-dominance-Z58
	670363597	pyruvate kinase, cytosolic isozyme	HP-dominance-Z58
	525342897	L-allo-threonine aldolase	HP-dominance-HD568
	293335401	uncharacterized protein LOC100279871	HP-dominance-HD568
	293333684	phospho-2-dehydro-3-deoxyheptonate aldolase 1	HP-dominance-Z58
	293332949	uncharacterized protein LOC100381786	HP-dominance-Z58
	293331181	hydroxymethylglutaryl-CoA synthase	HP-dominance-HD568
	226532381	glutamate synthase 1 [NADH], chloroplastic isoform X1	HP-dominance-Z58
	226532249	NADPH producing dehydrogenase of the oxidative pentose phosphate pathway	HP-dominance-Z58
	226530882	phosphoglucomutase, cytoplasmic 2	HP-dominance-Z58
	226529672	uncharacterized protein LOC100191846	HP-dominance-Z58
	226528689	dihydrodipicolinate synthase 2	HP-dominance-Z58
	226508708	ribulose-phosphate 3-epimerase	HP-dominance-Z58
	226506808	chorismate synthase 2	HP-dominance-HD568

226502865	uncharacterized protein LOC100286359	HP-dominance-HD568
226502522	6-phosphofructokinase	HP-dominance-Z58
226502058	6-phosphogluconate dehydrogenase 2	HP-dominance-HD568
226500284	hexokinase-3	HP-dominance-Z58
226499866	hexokinase-3	HP-dominance-HD568
226499532	fructose-bisphosphate aldolase cytoplasmic isozyme	HP-dominance-Z58
226499456	catalase isozyme 3	HP-dominance-Z58
226498808	cysteine synthase	HP-dominance-HD568
226497150	pyruvate dehydrogenase E1 component alpha subunit	HP-dominance-HD568
226496759	uncharacterized protein LOC100279587	HP-dominance-Z58
226495575	malate dehydrogenase 2	HP-dominance-HD568
226495363	uncharacterized protein LOC100273217	HP-dominance-HD568
226494977	uncharacterized protein LOC100383094 precursor	HP-dominance-Z58
226493858	PREDICTED: probable low-specificity L-threonine aldolase 2 isoform X3	HP-dominance-Z58
226493048	phosphoglucomutase, chloroplastic	HP-dominance-HD568
226492878	enolase isoform X1	HP-dominance-HD568
226491622	4-hydroxy-tetrahydrodipicolinate synthase, chloroplastic precursor	HP-dominance-Z58
219363693	glucose-6-phosphate 1-dehydrogenase, cytoplasmic isoform	HP-dominance-HD568
212275438	apospory-associated protein C	HP-dominance-HD568
212275278	uncharacterized LOC100280203	HP-dominance-Z58
212275097	3-dehydroquinate synthase	HP-dominance-Z58
162462912	triosephosphate isomerase, cytosolic	HP-dominance-Z58
162461460	uncharacterized protein LOC100382228	HP-dominance-HD568
162459678	D-3-phosphoglycerate dehydrogenase 3, chloroplastic	HP-dominance-Z58
162458262	2,3-bisphosphoglycerate-independent phosphoglycerate mutase isoform X1	HP-dominance-HD568
162457852	glycine dehydrogenase (decarboxylating), mitochondrial precursor	HP-dominance-HD568

Supplementary Table S6 Differentially expressed proteins under each pathway in overdominance category

Pathways	Protein ID	Annotation	Expression pattern
metabolism pathways	162461873	vte4 - vitamin E synthesis4	overdominance
	162463134	anthranilate synthase 2	overdominance
	212274981	acco35 - 1-aminocyclopropane-1-carboxylate oxidase35	overdominance
	212276284	Pyruvate kinase family protein	overdominance
	212721146	adenylate kinase putative expressed	overdominance
	212723156	Ubiquinol-cytochrome C reductase iron-sulfur subunit	overdominance
	219362415	ATPase subunit 1	overdominance
	226491860	Succinyl-CoA ligase alpha subunit	overdominance
	226492108	Glycosyl hydrolase family protein	overdominance
	226499578	bifunctional 3-dehydroquinate dehydratase	overdominance
	226499630	nad2 - NADH dehydrogenase2	overdominance
	226501354	hydroxymethylglutaryl-CoA synthase putative expressed	overdominance
	226502364	dla1 - dihydrolipoamide S-acetyltransferase1	overdominance
	226502554	phosphofructokinase putative expressed	overdominance
	226502662	uncharacterized LOC100191365	overdominance
	226507400	aco2 - aconitase2	overdominance
	226509220	phosphoesterase family protein putative expressed	overdominance
	226509860	cytidine deaminase 1	overdominance
	226528593	peroxidase 68 precursor	overdominance
	226530543	NADH dehydrogenase 1 alpha subcomplex subunit 9	overdominance
	226532421	pantothenate kinase 2	overdominance
	259490577	endochitinase A precursor	overdominance
	293332239	nucleotide diphosphate kinase 1	overdominance

	293334149	glucose-6-phosphate dehydrogenase 6	overdominance
	293336560	2 3-bisphosphoglycerate-independent phosphoglycerate mutase	overdominance
	293336655	CTP synthase family protein	overdominance
	525345228	UDP-N-acetylglucosamine diphosphorylase	overdominance
	670376570	acetate--CoA ligase ACS, chloroplastic/glyoxysomal-like	overdominance
	670389603	PREDICTED: glutamate synthase 1 [NADH], chloroplastic-like isoform X1	overdominance
	670395170	PREDICTED: acyl-coenzyme A oxidase 2, peroxisomal-like	overdominance
	670407310	PREDICTED: phosphoinositide phospholipase C 2-like	overdominance
	832626324	cf1 - camouflage1	overdominance
	902763242	cml1 - coumarate ligase1	overdominance
	926459513	glycerol-3-phosphosphate acyltransferase1	overdominance
	930697392	molybdopterin biosynthesis MoaE family protein	overdominance
	670386791	LOW QUALITY PROTEIN: ATP synthase subunit alpha, mitochondrial	overdominance
**biosynthesis of secondary metabolites**	162461873	vte4 - vitamin E synthesis4	overdominance
	162463134	anthranilate synthase component I-1 chloroplast precursor	overdominance
	212274981	1-aminocyclopropane-1-carboxylate oxidase 1Acc oxidase	overdominance
	212276284	Pyruvate kinase family protein	overdominance
	226491860	succinyl-CoA ligase alpha-chain 2	overdominance
	226492108	Glycosyl hydrolase family protein	overdominance
	226499578	bifunctional 3-dehydroquinate dehydratase/shikimate	overdominance
	226501354	hydroxymethylglutaryl-CoA synthase putative expressed	overdominance
	226502364	dla1 - dihydrolipoamide S-acetyltransferase1	overdominance
	226502554	phosphofructokinase putative expressed	overdominance
	226502662	uncharacterized protein LOC100280100	overdominance
	226507400	aco2 - aconitase2	overdominance

	226509220	phosphoesterase family protein putative expressed	overdominance
	226528593	peroxidase 68 precursor	overdominance
	293332239	nucleotide diphosphate kinase 1	overdominance
	293333808	3-ketoacyl-CoA synthase putative expressed	overdominance
	293334149	glucose-6-phosphate dehydrogenase 6	overdominance
	293336560	2 3-bisphosphoglycerate-independent phosphoglycerate mutase	overdominance
	670376570	acetate--CoA ligase ACS, chloroplastic/glyoxysomal-like	overdominance
	670389603	PREDICTED: glutamate synthase 1 [NADH], chloroplastic-like isoform X1	overdominance
	670395170	PREDICTED: acyl-coenzyme A oxidase 2, peroxisomal-like	overdominance
	832626324	cf1 - camouflage1	overdominance
	902763242	cml1 - coumarate ligase1	overdominance
	926459513	glycerol-3-phosphosphate acyltransferase1	overdominance

Supplementary Table S7 Differentially expressed proteins under each pathway in underdominance category

Pathways	Protein ID	Annotation	Expression pattern
**Ribosome biogenesis in eukaryotes**	226492268	uncharacterized protein LOC100274701	underdominance
	226497658	uncharacterized protein LOC100276745	underdominance
	226503861	small nucleolar ribonucleoprotein complex subunit	underdominance
	226507204	nucleolar GTP-binding protein 1	underdominance
	670405552	PREDICTED: RNA-binding protein 28-like	underdominance
	670405556	PREDICTED: single-stranded TG1-3 DNA-binding protein-like	underdominance
	670416542	nucleolar GTP-binding protein 1	underdominance
	670446680	transducin beta-like protein 3	underdominance

Supplementary Table S8 DEPs mapped to ear trait-related QTLs

Accession	Gene	Chr.	Position		QTL			Annotation	Patterns
					LPD	MPD	HPD		
226500034	GRMZM2G004298	1	66000310	66001719	*qLed1*	*qMed1*	*qHed1*	rhicadhesin receptor	HP-dominance-HD568
670363734	GRMZM2G113139	1	66581683	66586858	*qLed1*	*qMed1*	*qHed1*	formin-like protein 5	LP-dominance-Z58
924859523	GRMZM2G104353	1	67254188	67258645	*qLed1*	*qMed1*	*qHed1*	arginine/serine-rich splicing factor SC26 transcript III	LP-dominance-Z58
670361323	GRMZM2G107495	1	67437171	67439279	*qLed1*	*qMed1*	*qHed1*	uncharacterized protein	HP-dominance-Z58
212722526	GRMZM2G107473	1	67441969	67446297	*qLed1*	*qMed1*	*qHed1*	uncharacterized protein	HP-dominance-HD568
670358330	GRMZM2G077897	1	68568932	68572878	*qLed1*	*qMed1*	*qHed1*	uncharacterized protein	HP-dominance-HD568
293332317	GRMZM2G086920	2	4702643	4706609	***qLed2***			uncharacterized protein	LP-dominance-Z58
226499558	GRMZM2G033430	2	4839305	4841272	***qLed2***			uncharacterized protein	LP-dominance-Z58
670374829	GRMZM2G081886	2	5069598	5072692	***qLed2***			D-3-phosphoglycerate dehydrogenase, chloroplastic-like	HP-dominance-Z58
670374831	GRMZM2G082032	2	5073953	5075447	***qLed2***			L-asparaginase 2	over-dominance
670374833	GRMZM2G082097	2	5088192	5103724	***qLed2***			uncharacterized protein	LP-dominance-HD568
670374558	GRMZM2G160585	2	5235193	5239216	***qLed2***			glycosyltransferase	over-dominance
226491800	GRMZM2G126853	3	205338184	205340004	*qLed3-1*			uncharacterized protein	conserved
226499196	GRMZM2G157317	3	205695431	205702834	*qLed3-1*			uncharacterized protein	under-dominance
670389492	GRMZM2G122296	3	206225251	206228777	*qLed3-1*			phosphoethanolamine N-methyltransferase 1-like	ambiguous
670383571	GRMZM2G055450	3	206259530	206265981	*qLed3-1*			uncharacterized protein	HP-dominance-Z58
226509086	GRMZM2G146887	3	206834539	206835721	*qLed3-1*			Glutathione S-transferase GSTU6	conserved
670389524	GRMZM2G055807	3	206937314	206945700	*qLed3-1*			DNA repair helicase	HP-dominance-Z58

							XPB1-like	
670389548	GRMZM2G312712	3	207142766	207158874	*qLed3-1*		uncharacterized protein	under-dominance
226498812	GRMZM2G054715	3	208122523	208133257	*qLed3-1*		ATPase-containing subunit	conserved
670389603	GRMZM2G085078	3	208411907	208423612	***qLed3-1***		glutamate synthase 1	over-dominance
212276318	GRMZM2G150160	3	208492370	208496307	*qLed3-1*		uncharacterized protein	conserved
293331365	GRMZM2G152127	3	208546055	208552820	*qLed3-1*		putative AMP-dependent synthetase and ligase superfamily protein	conserved
226503922	GRMZM2G092152	3	208983735	208986596	*qLed3-1*		uncharacterized protein	HP-dominance-HD568
670389619	GRMZM2G423202	3	209050563	209053343	*qLed3-1*		uncharacterized protein	under-dominance
226491127	GRMZM2G080499	3	209796183	209812031	*qLed3-1*		Mitogen activated protein kinase kinase kinase-related	ambiguous
226502068	GRMZM2G134628	3	220928320	220934085	*qLed3-2*		Serine/threonine phosphatases, family 2C	HP-dominance-HD568
162463474	AC155377.1_FG001	3	220967145	220986200	*qLed3-2*		myosin 1	ambiguous
670392014	GRMZM2G149321	3	221319707	221325626	*qLed3-2*		uncharacterized protein	LP-dominance-HD568
212722818	GRMZM2G019501	3	221702938	221706856	*qLed3-2*		uncharacterized protein	HP-dominance-Z58
821159843	GRMZM2G071021	3	221771183	221775333	*qLed3-2*		cytosolic aldehyde dehydrogenase RF2C	HP-dominance-Z58
308044575	GRMZM2G042008	3	223175877	223177851	*qLed3-2*		uncharacterized protein	under-dominance
219363439	GRMZM2G072080	3	223777623	223781783	*qLed3-2*		uncharacterized protein	HP-dominance-Z58
226502258	GRMZM2G458095	3	223823835	223825699	*qLed3-2*		uncharacterized protein	over-dominance
670390238	GRMZM2G157588	3	223828065	223832159	*qLed3-2*		iron-sulfur cluster co-chaperone protein HscB, mitochondrial	under-dominance
293332027	GRMZM2G361398	3	224206634	224220693	*qLed3-2*		uncharacterized protein	LP-dominance-HD568

226502034	GRMZM2G339540	3	224250413	224255475	*qLed3-2*	putative leucine-rich repeat receptor-like protein kinase family protein	over-dominance
212720729	GRMZM2G115342	4	236304950	236308119	*qLed4*	uncharacterized protein	HP-dominance-Z58
212274831	GRMZM2G051004	4	236626967	236631977	*qLed4*	glyceraldehyde-3-phosphate dehydrogenase, cytosolic	LP-dominance-Z58
670392675	GRMZM2G168888	4	236927942	236931284	*qLed4*	uncharacterized protein	conserved
670392200	GRMZM2G119640	4	237433598	237440173	*qLed4*	zinc finger C-x8-C-x5-C-x3-H type family protein	HP-dominance-HD568
226528689	GRMZM2G154007	1	19326236	19329031	*qLel1*	alcohol dehydrogenase	HP-dominance-Z58
670362376	GRMZM2G002002	1	20282831	20291576	*qLel1*	uncharacterized protein	HP-dominance-Z58
293336229	GRMZM2G134367	1	20418111	20419920	*qLel1*	uncharacterized protein	HP-dominance-HD568
670391030	GRMZM2G111191	1	20989350	20994219	*qLel1*	uncharacterized protein	ambiguous
927028843	GRMZM2G411084	1	21081862	21082842	*qLel1*	MFP1 attachment factor 1	LP-dominance-HD568
670360084	GRMZM2G004259	1	21456080	21460930	*qLel1*	Mitochondrial ribosomal protein L51	LP-dominance-Z58
293331723	GRMZM2G049895	3	32034642	32037699	*qLel3*	uncharacterized protein	under-dominance
670386154	GRMZM2G070264	3	32245808	32251234	*qLel3*	DNA repair protein rhp26	over-dominance
670386175	GRMZM2G132936	3	32950294	32958127	*qLel3*	nipped-B-like protein B	HP-dominance-Z58
226507082	GRMZM2G134192	3	33773555	33777300	*qLel3*	uncharacterized protein	conserved
226494043	GRMZM2G178875	3	33795818	33797209	*qLel3*	uncharacterized protein	ambiguous
670386240	EF517601.1_FG015	3	34670044	34674766	*qLel3*	protein TRIGALACTOSYLDIACYL GLYCEROL 4, chloroplastic	conserved
226498092	GRMZM2G180065	3	35495835	35503247	*qLel3*	putative protein kinase superfamily protein	conserved

670386258	GRMZM2G091119	3	35660410	35666290	*qLel3*			importin subunit alpha-1a	LP-dominance-Z58
226501094	GRMZM2G134235	2	60341380	60343188	*qLern2*	*qMern2*	*qHMern2*	uncharacterized protein	HP-dominance-Z58
226507388	GRMZM2G089507	2	60391162	60395782	*qLern2*	*qMern2*	*qHMern2*	uncharacterized protein	conserved
670376570	GRMZM2G157269	2	60902602	60907977	*qLern2*	*qMern2*	*qHMern2*	acetate--CoA ligase ACS, chloroplastic/glyoxysomal-lik e	over-dominance
226500014	GRMZM2G069708	2	61285692	61289836	*qLern2*	*qMern2*	*qHMern2*	3-beta hydroxysteroid dehydrogenase/isomerase family protein	HP-dominance-Z58
930697382	GRMZM2G081652	3	173515706	173518871	*qLern3-1*	*qMern3-1*		uncharacterized protein	HP-dominance-Z58
226532636	GRMZM2G046846	3	173731626	173742437	*qLern3-1*	*qMern3-1*		uncharacterized protein	under-dominance
673921728	GRMZM2G097821	3	174102966	174108133	*qLern3-1*	*qMern3-1*		3-phosphoinositide-dependent protein kinase 2-like	ambiguous
670383793	GRMZM2G034985	3	224760341	224764489	*qLern3-2*	*qMern3-2*		uncharacterized protein	HP-dominance-HD568
226504174	GRMZM2G049866	3	225311288	225312863	*qLern3-2*	*qMern3-2*		heterogeneous nuclear ribonucleoprotein A3-like protein 2	LP-dominance-Z58
226494616	GRMZM2G094428	3	225426503	225429660	*qLern3-2*	*qMern3-2*		uncharacterized protein	conserved
670393431	GRMZM2G139760	4	208030243	208035544	*qLern4*	*qMern4*		hypothetical protein	HP-dominance-Z58
226509004	GRMZM2G060800	4	208419820	208426266	*qLern4*	*qMern4*		aldehyde dehydrogenase, dimeric NADP-preferring	ambiguous
670399382	GRMZM2G115245	4	208570971	208581479	*qLern4*	*qMern4*		DNA polymerase delta catalytic subunit	LP-dominance-Z58
939699111	GRMZM2G106424	4	209972408	209975654	*qLern4*	*qMern4*		14-3-3-like protein	LP-dominance-Z58
226501690	GRMZM2G002652	4	210865185	210868144	*qLern4*	*qMern4*		aminotransferase	ambiguous

670399429	GRMZM2G048274	4	210933399	210940029	*qLern4*	*qMern4*		hypothetical protein	LP-dominance-Z58
226500770	GRMZM2G104847	4	211465255	211473642	*qLern4*	*qMern4/qMed4*	*qHern4*	uncharacterized protein	HP-dominance-Z58
670418375	GRMZM2G111017	6	161851517	161854538	*qLknpr6*			probable ADP,ATP carrier protein	LP-dominance-Z58
226508766	GRMZM2G121237	6	161911254	161915727	*qLknpr6*			uncharacterized protein	HP-dominance-Z58
226528936	GRMZM5G847982	6	161998678	162000549	*qLknpr6*			uncharacterized protein	conserved
226532662	GRMZM2G096475	6	162162303	162163681	*qLknpr6*			lea protein group 3	over-dominance
670413509	GRMZM2G079617	6	162214972	162217495	*qLknpr6*			polygalacturonase	LP-dominance-Z58
670391769	GRMZM2G306255	6	162846251	162848017	*qLknpr6*			uncharacterized	ambiguous
226501502	GRMZM2G017269	6	162887447	162890818	*qLknpr6*			uncharacterized protein	conserved
162463564	GRMZM2G113216	6	163118403	163123973	*qLknpr6*			thiol oxidoreductase 1	ambiguous
670362755	GRMZM2G165944	1	33722785	33730720	*qLknr1*			filament-like plant protein 4	ambiguous
670371763	GRMZM2G045331	2	10486289	10491621	*qLknr2*		*qHel2*	Rcd1-like transcription factor	LP-dominance-Z58
212275816	GRMZM2G045596	2	10500994	10504096	*qLknr2*		*qHel2*	uncharacterized protein	LP-dominance-Z58
212274473	GRMZM2G046070	2	10537220	10541464	*qLknr2*		*qHel2*	cinnamyl alcohol dehydrogenase 1	HP-dominance-HD568
670375135	GRMZM2G093603	2	10562532	10567539	*qLknr2*		*qHel2*	uncharacterized protein	LP-dominance-HD568
670374544	GRMZM2G104546	2	173486913	173506976		*qMed2*		aspartate kinase homoserine dehydrogenase 2	LP-dominance-HD568
670378320	GRMZM2G138178	2	174739894	174753703		*qMed2*		uncharacterized protein	HP-dominance-Z58
226502046	GRMZM2G018189	3	181152799	181155106		*qMed3*		SLT1 protein	ambiguous
162460649	GRMZM2G426953	3	182095773	182101296		*qMed3*		uncharacterized protein	HP-dominance-Z58
670388839	GRMZM2G096470	3	182131715	182144018		*qMed3*		TBC1 domain family member 8B-like	HP-dominance-Z58
226492328	GRMZM2G155954	3	182643894	182647765		*qMed3*		uncharacterized protein	over-dominance

226502648	GRMZM2G014720	3	182952380	182955812	*qMed3*		BCL-2 binding anthanogene-1	under-dominance
670388878	GRMZM2G118363	3	184115247	184121770	*qMed3*		target of Myb protein 1-like	HP-dominance-Z58
226503211	GRMZM2G068151	3	184683407	184686981	*qMed3*		serine/threonine-protein kinase NAK	over-dominance
670388919	GRMZM2G050089	3	185812406	185817191	*qMed3*		putative auxin efflux carrier-like protein PINY	over-dominance
293336560	GRMZM2G003385	3	185890860	185895517	*qMed3*		2,3-bisphosphoglycerate-indep endent phosphoglycerate mutase	over-dominance
670384958	GRMZM2G009849	3	186230720	186250485	*qMed3*		uncharacterized protein	HP-dominance-Z58
212275454	GRMZM2G133213	3	186291885	186295766	*qMed3*		ferric-chelate reductase NADPH	HP-dominance-Z58
293335934	GRMZM5G885529	3	186454265	186458529	*qMed3*		uncharacterized protein	conserved
226498086	GRMZM2G054821	3	186490660	186496919	*qMed3*		uncharacterized protein	HP-dominance-Z58
670382975	GRMZM2G069024	3	186543524	186547440	*qMed3*		uncharacterized protein	conserved
670384970	GRMZM2G159134	3	186806019	186810898	*qMed3*		putative bZIP transcription factor superfamily protein	HP-dominance-HD568
293332115	GRMZM2G460860	3	186840260	186841796	*qMed3*		uncharacterized protein	HP-dominance-HD568
162459518	GRMZM2G139710	3	187087313	187090540	*qMed3*		uncharacterized protein	ambiguous
226495745	GRMZM2G445100	3	187107233	187112745	*qMed3*		uncharacterized protein	HP-dominance-Z58
212723266	GRMZM2G460406	4	217206578	217220877	*qMed4*	*qHern4*	uncharacterized protein	LP-dominance-Z58
670393457	GRMZM2G422464	4	218670827	218688798	*qMed4*	*qHern4*	hypothetical protein	HP-dominance-Z58
212274321	GRMZM2G042099	3	198590119	198607744	*qMel3*		uncharacterized protein	over-dominance
226506246	GRMZM2G042818	3	198661214	198665961	*qMel3*		3-hydroxyisobutyryl-CoA hydrolase/ catalytic	LP-dominance-Z58
670389318	GRMZM5G856653	3	199340491	199346889	*qMel3*		hexokinase-6	LP-dominance-Z58

670389325	GRMZM2G159105	3	199550852	199557018	*qMel3*	uncharacterized protein	ambiguous
226532590	GRMZM2G159171	3	199559581	199563532	*qMel3*	glutamate carboxypeptidase 2	HP-dominance-HD568
670383665	GRMZM2G005082	3	199694407	199713402	*qMel3*	uncharacterized protein	LP-dominance-Z58
226532381	GRMZM5G879882	3	199898628	199906875	*qMel3*	6-phosphofructokinase	HP-dominance-Z58
670385121	GRMZM2G111510	3	200107689	200115853	*qMel3*	diphosphonucleotide phosphatase 2	LP-dominance-Z58
226495575	GRMZM2G158562	3	200205518	200209697	*qMel3*	uncharacterized protein farnesyltranstransferase	HP-dominance-HD568
226529557	GRMZM2G031308	3	200237342	200241748	*qMel3*	uncharacterized protein	HP-dominance-Z58
212722808	GRMZM2G078756	3	200258553	200262717	*qMel3*	uncharacterized protein	HP-dominance-Z58
902967432	GRMZM2G069865	3	200493640	200502999	*qMel3*	uncharacterized protein	HP-dominance-HD568
226505472	GRMZM5G800586	3	200503799	200505886	*qMel3*	uncharacterized protein	HP-dominance-HD568
226510274	GRMZM2G176912	3	201266130	201267246	*qMel3*	uncharacterized protein	conserved
670389363	GRMZM2G014499	3	201299857	201304298	*qMel3*	uncharacterized protein	HP-dominance-HD568
670388548	GRMZM2G034639	3	171190834	171199550	*qMern3-1*	uncharacterized protein	HP-dominance-HD568
670388560	GRMZM2G135743	3	172093498	172099397	*qMern3-1*	UDP-glucuronate:xylan alpha-glucuronosyltransferase 1-like	under-dominance
226508726	GRMZM2G129444	3	172490574	172520783	*qMern3-1*	uncharacterized protein	HP-dominance-Z58
226531854	GRMZM2G120814	3	172609254	172617662	*qMern3-1*	snRK1-interacting protein 1	LP-dominance-Z58
670388638	GRMZM2G110897	3	174803532	174812741	*qMern3-1*	probable ion channel POLUX	over-dominance
162463509	GRMZM2G077333	3	174868242	174870828	*qMern3-1*	photosystem II subunit PsbS1	conserved
226529197	GRMZM2G056495	3	175324865	175330194	*qMern3-1*	uncharacterized protein	under-dominance
670384904	GRMZM2G141185	3	175391555	175417263	*qMern3-1*	uncharacterized protein	HP-dominance-Z58
162459002	GRMZM2G144653	7	13181810	13184354	*qMern7*	thioredoxin h homolog 2	under-dominance
670420415	GRMZM2G462325	7	19095051	19101243	*qMern7*	uncharacterized	conserved

162458781	GRMZM2G042627	7	19331382	19358462	*qMern7*	kinase associated protein phosphatase	LP-dominance-Z58
212721892	GRMZM2G038313	7	19501876	19506645	*qMern7*	uncharacterized protein	conserved
670422599	AC235546.1_FG001	7	19761619	19765853	*qMern7*	uncharacterized protein	ambiguous
670422603	GRMZM2G361615	7	19835645	19843639	*qMern7*	pentatricopeptide repeat-containing protein	HP-dominance-Z58
226508536	AC233961.1_FG001	7	20255014	20257584	*qMern7*	uncharacterized protein	LP-dominance-HD568
226503861	AC212570.3_FG006	7	20630441	20632054	*qMern7*	small nucleolar ribonucleoprotein complex subunit	under-dominance
226493125	GRMZM2G031780	7	21200138	21209290	*qMern7*	uncharacterized protein	LP-dominance-Z58
670422620	GRMZM2G165098	7	21431248	21433436	*qMern7*	nitrile-specifier protein 5	LP-dominance-Z58
219362877	GRMZM2G074549	7	23547847	23551271	*qMern7*	uncharacterized protein	HP-dominance-HD568
226506354	GRMZM2G010754	5	84974872	84980290	*qMknpr5*	splicing factor, arginine/serine-rich 12	LP-dominance-Z58
226491718	GRMZM2G098857	5	87156733	87160116	*qMknpr5*	uncharacterized protein	HP-dominance-Z58
293335299	GRMZM2G335638	5	87806330	87827981	*qMknpr5*	putative STRUBBELIG family receptor protein kinase	HP-dominance-Z58
226500226	GRMZM2G136599	5	88643464	88645956	*qMknpr5*	uncharacterized protein	ambiguous
902763242	GRMZM2G075333	5	89189678	89194264	*qMknpr5*	uncharacterized protein coumarate ligase1	over-dominance
226495659	GRMZM2G081144	5	90602005	90606551	*qMknpr5*	uncharacterized protein	LP-dominance-Z58
308081162	GRMZM2G472945	5	90798763	90801363	*qMknpr5*	TUB transcription factor	ambiguous
926657605	GRMZM2G360455	5	91844103	91851583	*qMknpr5*	protein phosphatase 2C isoform gamma	LP-dominance-Z58
226492100	GRMZM2G070015	5	93197666	93208356	*qMknpr5*	uncharacterized protein	HP-dominance-Z58

670402833	GRMZM2G060561	5	93267547	93273609	*qMknpr5*		uncharacterized protein	ambiguous
670420369	GRMZM2G107654	7	158175062	158191500	*qMknpr7*	*qHknpr7*	uncharacterized protein	LP-dominance-Z58
212275592	GRMZM2G107565	7	158194837	158199300	*qMknpr7*	*qHknpr7*	uncharacterized protein	HP-dominance-Z58
836467719	GRMZM2G006942	7	158498408	158506178	*qMknpr7*	*qHknpr7*	exocyst complex component EXO84C	HP-dominance-Z58
670414273	AC225308.2_FG005	7	158641922	158646713	*qMknpr7*	*qHknpr7*	probable ADP,ATP carrier protein At5g56450-like	HP-dominance-HD568
670425192	GRMZM2G380732	7	158965973	158971353	*qMknpr7*	*qHknpr7*	uncharacterized protein	conserved
226503221	GRMZM2G107562	7	159168808	159175289	*qMknpr7*	*qHknpr7*	uncharacterized protein	HP-dominance-Z58
226507620	GRMZM2G102514	7	160551419	160553078	*qMknpr7*	*qHknpr7*	protein BZR1 homolog 1-like	LP-dominance-HD568
226503529	GRMZM2G058087	7	160628660	160632132	*qMknpr7*	*qHknpr7*	ruvB-like 2	HP-dominance-HD568
212721462	GRMZM2G064437	7	161102509	161104652	*qMknpr7*	*qHknpr7*	proton myo-inositol cotransporter	HP-dominance-Z58
293335121	GRMZM2G098517	7	161325637	161329188	*qMknpr7*	*qHknpr7*	uncharacterized protein	HP-dominance-HD568
226493739	GRMZM2G398506	7	161329465	161340038	*qMknpr7*	*qHknpr7*	uncharacterized protein	LP-dominance-Z58
226502326	GRMZM2G396397	7	161724590	161732784	*qMknpr7*	*qHknpr7*	uncharacterized protein	LP-dominance-Z58
293331729	GRMZM2G151649	7	162043060	162044852	*qMknpr7*	*qHknpr7*	uncharacterized protein	over-dominance
226492142	GRMZM2G042683	1	65091947	65093876		*qHed1*	ribonucleoprotein A	LP-dominance-HD568
670363706	GRMZM2G121996	1	65490465	65494596		*qHed1*	uncharacterized protein	HP-dominance-Z58
212724102	GRMZM2G097426	1	65624367	65627202		*qHed1*	uncharacterized protein	HP-dominance-HD568
162459902	GRMZM2G048324	1	70432377	70436980		*qHed1*	nucleoredoxin 1	conserved
226502088	GRMZM2G139878	1	70729186	70731364		*qHed1*	GS3-like protein	LP-dominance-HD568
226509589	GRMZM2G083642	1	71344843	71347874		*qHed1*	small nuclear ribonucleoprotein G	LP-dominance-HD568
226529907	GRMZM2G036543	1	74274795	74287215		*qHed1*	uncharacterized protein	LP-dominance-HD568
293332009	GRMZM2G075892	1	76242264	76243725		*qHed1*	uncharacterized protein	conserved

670358706	GRMZM2G020523	1	77266302	77358639	*qHed1*	uncharacterized protein	over-dominance
670363962	GRMZM2G132794	1	77494290	77496510	*qHed1*	protein SHORT-ROOT 2-like	HP-dominance-Z58
670359285	GRMZM2G053946	1	77805748	77809482	*qHed1*	uncharacterized protein	HP-dominance-Z58
670363978	GRMZM2G050984	1	78709345	78715745	*qHed1*	uncharacterized protein	HP-dominance-Z58
836469121	GRMZM2G048821	1	79243435	79249210	*qHed1*	thymidine kinase	LP-dominance-Z58
670360817	GRMZM2G088793	1	79478032	79484427	*qHed1*	uncharacterized protein	over-dominance
670364010	GRMZM2G045090	1	79760351	79764442	*qHed1*	5-oxoprolinase	HP-dominance-Z58
670359729	GRMZM2G162690	1	80003683	80008544	*qHed1*	uncharacterized protein	HP-dominance-Z58
670360898	GRMZM2G064804	1	80065673	80093376	*qHed1*	uncharacterized protein	LP-dominance-Z58
293333882	GRMZM2G066441	1	81219487	81222936	*qHed1*	putative cytochrome P450 superfamily protein	LP-dominance-Z58
226499410	GRMZM2G162292	1	82584684	82587334	*qHed1*	uncharacterized protein	LP-dominance-HD568
226491414	GRMZM2G026147	1	85027909	85029759	*qHed1*	expansin-like 3	conserved
162457743	GRMZM2G175134	1	85725085	85726426	*qHed1*	glutathione transferase14	LP-dominance-Z58
670358653	GRMZM2G162537	1	87631542	87636449	*qHed1*	uncharacterized protein	conserved
670439107	GRMZM2G079487	1	88539374	88552173	*qHed1*	uncharacterized protein	LP-dominance-Z58
670360893	GRMZM2G107741	1	88896254	88908802	*qHed1*	uncharacterized protein	LP-dominance-Z58
293331453	GRMZM5G813892	1	89448263	89451835	*qHed1*	putative MYB DNA-binding domain superfamily protein	HP-dominance-Z58
673921632	GRMZM2G021621	1	91256138	91258958	*qHed1*	expansin-B4	conserved
670364256	GRMZM2G082916	1	91776842	91782024	*qHed1*	ARF guanine-nucleotide exchange factor GNOM-like	over-dominance
931716303	GRMZM2G052666	1	94495828	94498454	*qHed1*	uncharacterized protein	HP-dominance-HD568
927261937	GRMZM2G180909	1	94881328	94884787	*qHed1*	nucleic acid binding protein	HP-dominance-Z58
226533252	GRMZM2G481163	1	94888973	94890564	*qHed1*	trihelix transcription factor	over-dominance
219363405	GRMZM2G180916	1	94897526	94901091	*qHed1*	SnRK2.3	HP-dominance-Z58

308080602	GRMZM2G086869	1	96691323	96693175	*qHed1*	uncharacterized protein	LP-dominance-Z58
670364306	AC211390.3_FG001	1	97042658	97047761	*qHed1*	uncharacterized protein	conserved
670358670	GRMZM2G001895	1	98344349	98360337	*qHed1*	uncharacterized protein	over-dominance
670364316	GRMZM2G001930	1	98363081	98365897	*qHed1*	myc transcription factor 7	over-dominance
212724002	GRMZM2G169931	1	99137239	99139419	*qHed1*	uncharacterized protein	conserved
226494131	GRMZM2G024823	1	99180327	99184120	*qHed1*	uncharacterized protein	conserved
226499154	AC194914.3_FG002	1	99547306	99547944	*qHed1*	cyclase/dehydrase family protein	LP-dominance-Z58
226531494	GRMZM2G160454	1	100591387	100605965	*qHed1*	uncharacterized protein	under-dominance
162459062	GRMZM2G456626	1	101838959	101841220	*qHed1*	low phytic acid 2	ambiguous
212723850	GRMZM2G457147	1	105335718	105337440	*qHed1*	uncharacterized protein	HP-dominance-Z58
670364432	GRMZM2G128641	1	105768302	105774413	*qHed1*	uncharacterized proteinprotein RSN1	HP-dominance-Z58
226499624	GRMZM2G321940	1	107452921	107458739	*qHed1*	uncharacterized protein	over-dominance
670360663	GRMZM2G159992	1	107890804	107901599	*qHed1*	uncharacterized protein	ambiguous
226505384	GRMZM2G041613	1	109394550	109444450	*qHed1*	uncharacterized protein	HP-dominance-Z58
670358250	GRMZM2G083475	1	112148430	112156004	*qHed1*	uncharacterized protein	LP-dominance-HD568
212274747	GRMZM2G015767	1	112734296	112741865	*qHed1*	PREDICTED: ATPase family AAA domain-containing protein 1 isoform X1	conserved
670364568	GRMZM2G002515	1	113534938	113538490	*qHed1*	serine/threonine-protein kinase BRI1-like 2	HP-dominance-Z58
226494686	GRMZM2G018595	2	1437710	1441525	*qHed2*	uncharacterized protein	HP-dominance-Z58
226529753	GRMZM2G461145	5	9643635	9648187	*qHed5*	uncharacterized protein	ambiguous
308080672	GRMZM2G093139	5	10020204	10022746	*qHed5*	uncharacterized protein	HP-dominance-HD568
670405872	GRMZM2G181002	5	10095299	10102902	*qHed5*	uncharacterized protein	LP-dominance-HD568

226529707	GRMZM2G425236	5	10223283	10224485	*qHed5*	ZF-HD protein dimerisation region containing protein	LP-dominance-Z58
670405904	GRMZM2G158359	5	10425114	10429156	*qHed5*	probable receptor protein kinase TMK1	over-dominance
670403299	GRMZM2G114789	5	10552445	10556342	*qHed5*	uncharacterized protein	HP-dominance-Z58
212276092	GRMZM2G131324	5	10594380	10598789	*qHed5*	ATP binding protein	LP-dominance-Z58
226498594	GRMZM2G150448	5	10907886	10911422	*qHed5*	atypical receptor-like kinase MARK precursor	LP-dominance-HD568
670404014	GRMZM2G090172	5	10941559	10947265	*qHed5*	uncharacterized protein	under-dominance
670405932	GRMZM2G114162	5	11354693	11382200	*qHed5*	phosphatidylinositol 4-kinase alpha 1-like	HP-dominance-Z58
226505874	GRMZM2G177942	5	11495276	11497686	*qHed5*	uncharacterized protein	LP-dominance-Z58
226499432	GRMZM2G177934	5	11500765	11501671	*qHed5*	chemocyanin	over-dominance
226505952	GRMZM2G177885	5	11502314	11506400	*qHed5*	uncharacterized protein	LP-dominance-Z58
226510036	GRMZM2G123459	5	11614767	11617391	*qHed5*	ATP-dependent RNA helicase DDX23	HP-dominance-Z58
219363289	GRMZM2G102754	5	12279143	12285202	*qHed5*	uncharacterized protein	conserved
162463192	GRMZM2G102760	5	12285656	12290564	*qHed5*	lipoxygenase	HP-dominance-Z58
226498974	AC206941.2_FG002	5	12302727	12303287	*qHed5*	oleosin 18 kDa	ambiguous
226532014	GRMZM2G111566	5	12576565	12579887	*qHed5*	proteasome component 2	LP-dominance-Z58
226498438	GRMZM2G076524	5	13264659	13269094	*qHed5*	uncharacterized protein	HP-dominance-HD568
226529836	GRMZM2G115156	9	21725714	21732320	*qHed9/qHel9*	uncharacterized protein	under-dominance
670437428	GRMZM2G447455	9	21886129	21901757	*qHed9/qHel9*	uncharacterized protein	LP-dominance-HD568
226503097	GRMZM2G097313	9	22489480	22493454	*qHed9/qH*	uncharacterized protein	LP-dominance-HD568

					el9		
670435142	GRMZM2G092296	9	22666627	22668837	*qHed9/qHel9*	uncharacterized protein	ambiguous
670437449	GRMZM5G877500	9	22689315	22692638	*qHed9/qHel9*	3-phosphoshikimate 1-carboxyvinyltransferase	conserved
670435780	GRMZM2G082855	9	24074354	24081173	*qHed9/qHel9*	putative leucine-rich repeat receptor-like protein kinase family protein	HP-dominance-Z58
293336604	GRMZM2G350319	9	24481802	24487428	*qHed9/qHel9*	uncharacterized protein	ambiguous
670435916	GRMZM2G048557	9	24705511	24710303	*qHed9/qHel9*	39S ribosomal protein L47	LP-dominance-Z58
212275085	GRMZM5G871262	9	24959346	24964546	*qHed9/qHel9*	uncharacterized protein	ambiguous
670437538	GRMZM2G119411	9	25726920	25741438	*qHed9/qHel9*	endoplasmic reticulum metallopeptidase 1	HP-dominance-Z58
948284257	GRMZM2G042080	9	25828876	25831557	*qHed9/qHel9*	superoxide dismutase	LP-dominance-HD568
819231707	GRMZM2G136838	9	26591337	26597907	*qHed9/qHel9*	kinesin-like protein 2	LP-dominance-HD568
226493211	GRMZM2G051890	9	26631544	26635096	*qHed9/qHel9*	uncharacterized protein	HP-dominance-HD568
226499874	GRMZM2G475293	9	26831058	26833409	*qHed9/qHel9*	uncharacterized protein	LP-dominance-Z58
670444809	GRMZM2G377369	4	26710769	26715748	*qHel4*	uncharacterized protein	LP-dominance-HD568
670393682	GRMZM2G093900	4	26919963	26927993	*qHel4*	aminotransferase y4uB	HP-dominance-HD568

226530577	GRMZM2G129615	4	28858321	28861914	*qHel4*	uncharacterized protein	LP-dominance-HD568
226504376	GRMZM2G031529	10	141370294	141376285	*qHern10*	uncharacterized protein	under-dominance
670372868	GRMZM2G028676	10	141395829	141397031	*qHern10*	uncharacterized protein	LP-dominance-Z58
525345228	GRMZM2G019986	10	141495963	141500441	*qHern10*	UDP-N-acetylglucosamine diphosphorylase	over-dominance
670443736	GRMZM2G025243	10	141600894	141612028	*qHern10*	peroxisomal 2,4-dienoyl-CoA reductase-like	LP-dominance-HD568
162463785	GRMZM2G025592	10	141612636	141620390	*qHern10*	DNA cytosine methyltransferase MET2a	under-dominance
670448133	GRMZM2G114182	10	141754578	141761451	*qHern10*	probable polyamine oxidase 2	HP-dominance-HD568
670446774	GRMZM2G354867	10	141823070	141828449	*qHern10*	protein argonaute 2-like	HP-dominance-Z58
162459783	GRMZM2G121612	10	142347313	142356351	*qHern10*	starch synthase IIIb-1 precursor	LP-dominance-Z58
670446831	GRMZM2G396856	10	142484991	142490205	*qHern10*	probable polyamine oxidase 2	LP-dominance-Z58
670443262	GRMZM2G396846	10	142492885	142494888	*qHern10*	bromodomain containing protein	HP-dominance-Z58
212722596	GRMZM2G058098	10	142670634	142675088	*qHern10*	uncharacterized protein	under-dominance
670446850	GRMZM2G148506	10	142876623	142885325	*qHern10*	uncharacterized protein	conserved
670446857	GRMZM2G148594	10	142914867	142924821	*qHern10*	uncharacterized protein	HP-dominance-Z58
827834142	GRMZM2G180471	10	143089757	143102257	*qHern10*	uncharacterized protein	over-dominance
226531123	GRMZM5G895313	10	143487131	143488239	*qHern10*	uncharacterized protein	LP-dominance-Z58
670390861	GRMZM2G303661	3	30541433	30545499	*qHern3*	uncharacterized protein	LP-dominance-HD568

[a] QTL, q + planting density abbreviation + trait abbreviation + chromosome number + QTL number, e.g., *qLed3-1*,corresponds to the first QTL for ear diameter on chromosome 3 under low planting density.

[b] LPD, low planting density; MPD, Medium planting density; HPD, high planting density.

Supplementary Table S9 DEPs mapped to kernel trait-related QTLs

Accession	Gene	Chr.	Position		QTL			Annotation	Patterns
					LPD	MPD	HPD		
226497062	GRMZM2G049041	1	38254352	38257177	*qLhkw1*	*qMhkw1*	*qHhkw1*	uncharacterized protein	HP-dominance-HD568
226499192	GRMZM2G160906	1	40730452	40733267	*qLhkw1*	*qMhkw1*	*qHhkw1*	putative ENTH/ANTH/VHS superfamily protein3 clathrin assembly protein putative expressed	HP-dominance-Z58
226501908	GRMZM2G540403	1	38320998	38327847	*qLhkw1*	*qMhkw1*	*qHhkw1*	50S ribosomal protein L4	HP-dominance-Z58
226506884	GRMZM2G428410	1	41108326	41111313	*qLhkw1*	*qMhkw1*	*qHhkw1*	uncharacterized protein	LP-dominance-Z58
226507514	GRMZM2G141799	1	39217739	39224044	*qLhkw1*	*qMhkw1*	*qHhkw1*	uncharacterized protein	HP-dominance-HD568
226530284	GRMZM2G128206	1	38621379	38623448	*qLhkw1*	*qMhkw1*	*qHhkw1*	pollenspecific protein SF	HP-dominance-Z58
226530894	GRMZM2G087459	1	40426197	40429337	*qLhkw1*	*qMhkw1*	*qHhkw1*	protein kinase APK1A	HP-dominance-Z58
293337139	GRMZM2G001918	1	39736525	39741619	*qLhkw1*	*qMhkw1*	*qHhkw1*	uncharacterized protein	LP-dominance-HD568
670359042	GRMZM2G012319	1	38540863	38552452	*qLhkw1*	*qMhkw1*	*qHhkw1*	uncharacterized protein	HP-dominance-Z58
670359386	GRMZM2G160452	1	39072122	39077770	*qLhkw1*	*qMhkw1*	*qHhkw1*	digalactosyldiacylglycerol synthase 2 chloroplastic	HP-dominance-Z58
670360881	GRMZM2G109885	1	39852701	39860136	*qLhkw1*	*qMhkw1*	*qHhkw1*	uncharacterized protein	HP-dominance-HD568

670362952	GRMZM2G145275	1	41221754	41225729	*qLhkw1*	*qMhkw1*	*qHhkw1*	heat shock cognate 70 kDa proteinlike	conserved
670362965	GRMZM2G044281	1	41484792	41488160	*qLhkw1*	*qMhkw1*	*qHhkw1*	probable rhamnose biosynthetic enzyme 1	conserved
931716140	GRMZM2G174644	1	40226665	40231221	*qLhkw1*	*qMhkw1*	*qHhkw1*	uncharacterized protein	
212722732	GRMZM6G910222	4	239674972	239676894	*qLhkw4*			uncharacterized protein	LP-dominance-Z58
226498032	GRMZM2G090156	4	238707295	238712177	*qLhkw4*			uncharacterized protein	LP-dominance-Z58
670392416	GRMZM2G010044	4	239420850	239424772	*qLhkw4*			uncharacterized protein	conserved
670400277	GRMZM2G019106	4	239534487	239538429	*qLhkw4*			transcription factor HBP1alike	HP-dominance-HD568
670400321	GRMZM5G878541	4	239984257	239988267	*qLhkw4*			uncharacterized protein	conserved
818213455	AC233922.1_FG005	4	238598643	238601466	*qLhkw4*			bifunctional protein FolD 4, chloroplastic	LP-dominance-Z58
226506654	GRMZM2G056075	7	162805707	162815421	*qLhkw7*			MutS homolog 1	LP-dominance-HD568
293334661	GRMZM2G090715	7	162456979	162470174	*qLhkw7*			uncharacterized protein	HP-dominance-HD568
308081401	GRMZM2G092752	7	162361838	162363739	*qLhkw7*			uncharacterized protein	over-dominance
922959999	GRMZM2G181219	7	162381461	162383864	*qLhkw7*			uncharacterized protein	conserved
930944872	GRMZM2G053338	7	162709132	162711616	*qLhkw7*			indole3acetic acidamido synthetase GH3.8	under-dominance
162457932	GRMZM2G107867	1	44371887	44378439	*qLkl1-1*	*qMkl1-1*	*qHkl1-1/ qHkw1*	SNF1related protein kinase	HP-dominance-Z58

162462104	GRMZM2G073628	1	44196096	44197734	*qLkl1-1*	*qMkl1-1*	*qHkl1-1/ qHkw1*	protein disulfide isomerase	LP-dominance-Z58
162460824	GRMZM2G148281	1	207946160	207950205	*qLkl1-2*			uncharacterized protein	LP-dominance-HD568
670405786	GRMZM2G312738	5	8185472	8196608	*qLkl5*			E3 ubiquitinprotein ligase HOS1like	HP-dominance-Z58
212274887	GRMZM2G031311	6	133839861	133843446	*qLkt6*			uncharacterized protein	HP-dominance-Z58
226508688	GRMZM2G055678	6	134648617	134656424	*qLkt6*			uncharacterized protein	over-dominance
670412456	GRMZM2G088501	6	134052641	134061915	*qLkt6*			uncharacterized protein	over-dominance
670419608	GRMZM6G314950	6	134480025	134482855	*qLkt6*			DNA primase small subunit	under-dominance
226490910	GRMZM2G088843	1	29741944	29744117	*qLkw1*			uncharacterized protein	HP-dominance-HD568
226491696	GRMZM2G002178	1	28926619	28928612	*qLkw1*			uncharacterized protein	HP-dominance-Z58
226499098	GRMZM2G043584	1	30258404	30262357	*qLkw1*			uncharacterized protein	conserved
226503653	GRMZM2G012501	1	30758125	30767310	*qLkw1*			uncharacterized protein	HP-dominance-Z58
293331791	GRMZM2G103579	1	28794281	28800513	*qLkw1*			uncharacterized protein	under-dominance
670358019	GRMZM2G116846	1	31246755	31249123	*qLkw1*			uncharacterized protein	HP-dominance-Z58
162463469	GRMZM2G098520	4	190228051	190230492	*qLkw4*		*qHhkw4*	ribulose bisphosphate carboxylase small subunit 1	HP-dominance-Z58
618465360	GRMZM2G033785	4	190849608	190860185	*qLkw4*		*qHhkw4*	uncharacterized protein	LP-dominance-HD568
226494917	GRMZM2G115499	4	185109363	185112676		*qMhkw4*		uncharacterized protein	LP-dominance-HD568
670398647	GRMZM2G103281	4	185354844	185359629		*qMhkw4*		ethanolaminephosphate	LP-dominance-Z58

							cytidylyltransferaselike	
226507661	GRMZM2G129554	5	9183837	9194614	*qMkl5*	*qHkl5*	uncharacterized protein	LP-dominance-Z58
226508254	GRMZM2G129700	5	9198351	9202643	*qMkl5*	*qHkl5*	uncharacterized protein	HP-dominance-Z58
293336437	GRMZM2G521844	5	8510741	8515737	*qMkl5*	*qHkl5*	uncharacterized protein	HP-dominance-Z58
670405816	GRMZM2G064042	5	8555544	8557863	*qMkl5*	*qHkl5*	uncharacterized protein	LP-dominance-Z58
226505218	GRMZM2G054115	3	202790244	202793120	*qMkt3*	*qHkt3*	uncharacterized protein	LP-dominance-Z58
226508416	GRMZM2G069229	3	202208289	202210849	*qMkt3*	*qHkt3*	uncharacterized protein	under-dominance
226509860	GRMZM2G380088	3	203823955	203825474	*qMkt3*	*qHkt3*	Cytidine deaminase 1	over-dominance
670383378	AC207628.4_FG01 1	3	202916872	202920796	*qMkt3*	*qHkt3*	uncharacterized protein	HP-dominance-Z58
670389403	GRMZM5G863596	3	203014805	203037600	*qMkt3*	*qHkt3*	alphaamylase 3, chloroplastic	HP-dominance-Z58
670389410	GRMZM2G474537	3	203356767	203361639	*qMkt3*	*qHkt3*	uncharacterized protein	HP-dominance-Z58
670389426	GRMZM2G008764	3	204353127	204355837	*qMkt3*	*qHkt3*	protein IQDOMAIN 1like	LP-dominance-HD568
212275346	GRMZM2G111324	1	27626870	27631651	*qMkw1*		putative O-Glycosyl hydrolase superfamily protein	
293333616	GRMZM2G439311	4	188375246	188379448	*qMkw4*		putative calmodulinbinding family protein	LP-dominance-HD568
670392133	GRMZM2G011173	4	189215330	189219182	*qMkw4*		uncharacterized protein	
670392621	GRMZM2G006252	4	188159850	188168084	*qMkw4*		DNA binding protein	HP-dominance-Z58
670398775	GRMZM2G433528	4	188579929	188582253	*qMkw4*		hypothetical protein	LP-dominance-HD568

212722276	GRMZM2G043295	9	130786653	130788537	*qHhkw9*	anthocyanidin 5,3-O-glucosyltransferase	
219363191	GRMZM2G436092	9	130745221	130750075	*qHhkw9*	ASF/SF2like premRNA splicing factor SRP32	conserved
226494728	GRMZM2G134107	9	130759499	130760774	*qHhkw9*	uncharacterized protein	
226528531	GRMZM2G012970	9	131180228	131182741	*qHhkw9*	uncharacterized protein	under-dominance
670439574	GRMZM2G058522	9	129535000	129538698	*qHhkw9*	superoxide dismutase [CuZn] 4AP	conserved
670439644	GRMZM2G006080	9	131829772	131832943	*qHhkw9*	receptor like protein kinase FERONIA	HP-dominance-Z58
670441649	GRMZM2G007922	9	129218954	129226936	*qHhkw9*	DNA helicase INO80-like	under-dominance
11467187	GRMZM2G458118	9	61627828	61630396	*qHkl9*	ATPase III subunit	ambiguous
162459168	GRMZM2G168143	9	27028016	27030404	*qHkl9*	ferredoxin	LP-dominance-Z58
162459933	GRMZM2G013002	9	116265519	116268415	*qHkl9*	uncharacterized protein	conserved
162460681	GRMZM2G152908	9	122479052	122485725	*qHkl9*	sucrose synthase 1	conserved
162461720	GRMZM2G117935	9	95582500	95583611	*qHkl9*	superal 1	LP-dominance-Z58
162462687	GRMZM2G113873	9	118704079	118708998	*qHkl9*	cystathionine gammasynthase 1	conserved
162462912	GRMZM2G027835	9	107331165	107334148	*qHkl9*	dihydrodipicolinate synthase 1	HP-dominance-Z58
163838760	GRMZM2G400167	9	77335866	77339429	*qHkl9*	uncharacterized protein	LP-dominance-Z58
212275085	GRMZM5G871262	9	24959346	24964546	*qHkl9*	uncharacterized protein	

212275161	GRMZM2G134382	9	47658344	47660713	*qHkl9*	uncharacterized protein	over-dominance
212275324	GRMZM2G113873	9	118704079	118708998	*qHkl9*	uncharacterized protein	conserved
212720865	GRMZM2G144610	9	37552323	37553942	*qHkl9*	endosperm specific protein 1	conserved
212721020	GRMZM2G124317	9	123666928	123669967	*qHkl9*	uncharacterized protein	LP-dominance-Z58
212721110	GRMZM2G118873	9	38798325	38799842	*qHkl9*	uncharacterized protein	over-dominance
212721542	GRMZM2G148180	9	114301799	114306095	*qHkl9*	uncharacterized protein	HP-dominance-Z58
212722700	GRMZM2G139973	9	96451513	96457764	*qHkl9*	uncharacterized protein	LP-dominance-Z58
219363197	GRMZM2G033029	9	41210743	41217493	*qHkl9*	uncharacterized protein	over-dominance
226490831	GRMZM2G347931	9	106302354	106306175	*qHkl9*	uncharacterized protein	under-dominance
226490924	GRMZM2G081474	9	84342406	84348561	*qHkl9*	uncharacterized protein	under-dominance
226491021	GRMZM2G026952	9	116972384	116980458	*qHkl9*	uncharacterized protein	LP-dominance-Z58
226492989	GRMZM2G134045	9	112127022	112137834	*qHkl9*	ADP-ribosylation factor GTPaseactivating protein 3	HP-dominance-HD568
226493211	GRMZM2G051890	9	26631544	26635096	*qHkl9*	uncharacterized protein	HP-dominance-HD568
226494183	GRMZM2G058336	9	90204394	90205681	*qHkl9*	uncharacterized protein	conserved
226494253	GRMZM2G370155	9	24344507	24346244	*qHkl9*	uncharacterized protein	under-dominance
226494506	GRMZM2G009995	9	89736160	89741950	*qHkl9*	uncharacterized protein	HP-dominance-HD568
226494945	AC217887.3_FG005	9	122191826	122195365	*qHkl9*	premRNA splicing factor PRP17	HP-dominance-Z58

226495279	GRMZM2G142735	9	103704013	103706095	*qHkl9*	uncharacterized protein	LP-dominance-Z58
226496061	GRMZM2G056078	9	66294188	66330178	*qHkl9*	uncharacterized protein	LP-dominance-Z58
226499726	GRMZM2G009724	9	110588388	110590056	*qHkl9*	speckletype POZ protein	HP-dominance-Z58
226499874	GRMZM2G475293	9	26831058	26833409	*qHkl9*	uncharacterized protein	LP-dominance-Z58
226500010	GRMZM2G018619	9	77764194	77768544	*qHkl9*	uncharacterized protein	LP-dominance-Z58
226502036	GRMZM2G119146	9	115351180	115354729	*qHkl9*	nucleic acid binding protein	LP-dominance-Z58
226503181	GRMZM2G090849	9	109688338	109706995	*qHkl9*	cell division control protein 50	HP-dominance-Z58
226505518	GRMZM2G140082	9	96460028	96463796	*qHkl9*	protein kinase domain containing protein	HP-dominance-HD568
226507116	GRMZM2G031941	9	78315660	78317440	*qHkl9*	uncharacterized protein	over-dominance
226509158	GRMZM2G085932	9	46534726	46538538	*qHkl9*	uncharacterized protein	LP-dominance-Z58
226509422	GRMZM2G148702	9	89654357	89658343	*qHkl9*	uncharacterized protein	conserved
226528252	GRMZM2G000219	9	120503782	120505559	*qHkl9*	coppertransporting ATPase PAA1	LP-dominance-Z58
226528671	GRMZM2G469898	9	65038789	65041856	*qHkl9*	esterase	over-dominance
226530007	GRMZM2G170927	9	95183056	95188412	*qHkl9*	uncharacterized protein	HP-dominance-HD568
226530064	GRMZM2G131667	9	112904292	112906676	*qHkl9*	uncharacterized protein	LP-dominance-Z58
226530136	GRMZM2G352607	9	44243565	44250275	*qHkl9*	lipid binding protein	
226530207	GRMZM5G839512	9	91972448	91976378	*qHkl9*	uncharacterized protein	

226530470	GRMZM2G073123	9	40352961	40359027	*qHkl9*	uncharacterized protein	LP-dominance-Z58
226531193	GRMZM5G863602	9	106259080	106262078	*qHkl9*	senescenceassociated protein DH	LP-dominance-Z58
226533562	GRMZM2G442551	9	90709327	90713727	*qHkl9*	phospholipase D family protein	HP-dominance-Z58
259490629	GRMZM2G326472	9	93922466	93928074	*qHkl9*	uncharacterized protein	HP-dominance-Z58
293331149	GRMZM2G000566	9	102396762	102400757	*qHkl9*	uncharacterized protein	over-dominance
293332934	GRMZM2G136538	9	121844283	121848798	*qHkl9*	uncharacterized protein	under-dominance
293333358	GRMZM2G138987	9	123855368	123859064	*qHkl9*	uncharacterized protein	LP-dominance-HD568
293333482	GRMZM2G055320	9	107810217	107816811	*qHkl9*	uncharacterized protein	
293334967	GRMZM2G103236	9	113842221	113844729	*qHkl9*	uncharacterized protein	
293335415	GRMZM2G158595	9	45486736	45493452	*qHkl9*	uncharacterized protein	
293335981	GRMZM2G052389	9	28517992	28523621	*qHkl9*	uncharacterized protein	
293336489	GRMZM2G152105	9	122773640	122779565	*qHkl9*	uncharacterized protein	LP-dominance-Z58
293336604	GRMZM2G350319	9	24481802	24487428	*qHkl9*	uncharacterized protein	
308080628	GRMZM2G134844	9	112972499	112974551	*qHkl9*	uncharacterized protein	
670434982	GRMZM2G095206	9	89593771	89596984	*qHkl9*	uncharacterized protein	conserved
670434986	AC225193.3_FG003	9	113078265	113081316	*qHkl9*	uncharacterized protein	
670435065	GRMZM2G162968	9	124293005	124299258	*qHkl9*	uncharacterized protein	HP-dominance-HD568

670435107	GRMZM2G432722	9	105888217	105892082	*qHkl9*	uncharacterized protein	over-dominance
670435204	GRMZM2G093880	9	114801181	114843375	*qHkl9*	palmitoylprotein thioesterase 1	LP-dominance-Z58
670435349	GRMZM2G047564	9	88467925	88481742	*qHkl9*	uncharacterized protein	HP-dominance-Z58
670435479	GRMZM2G148216	9	114324162	114328478	*qHkl9*	uncharacterized protein	conserved
670435665	GRMZM2G105167	9	95249332	95254113	*qHkl9*	uncharacterized protein	LP-dominance-Z58
670435848	GRMZM2G087712	9	95009735	95019179	*qHkl9*	uncharacterized protein	under-dominance
670435916	GRMZM2G048557	9	24705511	24710303	*qHkl9*	39S ribosomal protein L47	LP-dominance-Z58
670436270	GRMZM2G096184	9	31100539	31112675	*qHkl9*	uncharacterized protein	under-dominance
670436288	GRMZM2G085246	9	106534395	106542631	*qHkl9*	putative STRUBBE	over-dominance
670436298	GRMZM2G151734	9	109707159	109710291	*qHkl9*	uncharacterized protein	
670436306	GRMZM2G151387	9	119964620	119969145	*qHkl9*	uncharacterized protein	HP-dominance-Z58
670436443	GRMZM2G443953	9	88990187	89015673	*qHkl9*	putative coatomer beta subunit family protein	conserved
670436451	GRMZM2G093065	9	95802810	95811159	*qHkl9*	uncharacterized protein	conserved
670436557	AC189879.3_FG003	9	87408375	87414276	*qHkl9*	uncharacterized protein	LP-dominance-Z58
670436698	GRMZM2G152908	9	122479052	122485725	*qHkl9*	sucrose synthase 1	LP-dominance-Z58
670437538	GRMZM2G119411	9	25726920	25741438	*qHkl9*	endoplasmic reticulum metallopeptidase 1	HP-dominance-Z58
670437849	GRMZM2G047178	9	44121275	44128502	*qHkl9*	vacuolar protein	conserved

						sortingassociated protein VTA1 homolog	
670438260	GRMZM2G041732	9	67914868	67934818	*qHkl9*	DEAD-box ATPdependent RNA helicase 31like	LP-dominance-Z58
670438437	GRMZM2G156818	9	82306386	82318831	*qHkl9*	RNA polymerase IIassociated protein 1	conserved
670438604	GRMZM2G074037	9	92413442	92420203	*qHkl9*	26S protease regulatory subunit 10B homolog A	LP-dominance-Z58
670438640	GRMZM2G348666	9	94453571	94475198	*qHkl9*	isoleucine--tRNA ligase, cytoplasmic [Zea mays]	conserved
670438657	GRMZM2G087806	9	95033444	95044390	*qHkl9*	protein Sacyltransferase 24like	HP-dominance-Z58
670438685	GRMZM2G141288	9	97192454	97196465	*qHkl9*	probable LRR receptorlike serine/threonineprotein kinase	HP-dominance-Z58
670438715	GRMZM2G130905	9	99285720	99289868	*qHkl9*	uncharacterized protein	
670438901	GRMZM5G836827	9	106897213	106902894	*qHkl9*	villin4like	
670438904	GRMZM2G028254	9	107334980	107337107	*qHkl9*	transmembrane protein 230like	over-dominance
670439016	GRMZM2G331105	9	114069436	114088947	*qHkl9*	uncharacterized protein	HP-dominance-HD568
670439046	GRMZM2G040991	9	115075691	115078291	*qHkl9*	uncharacterized protein	LP-dominance-Z58
670439186	GRMZM2G126120	9	119190370	119195584	*qHkl9*	KH domain-containing protein	over-dominance
670439300	GRMZM2G104676	9	121983392	121989352	*qHkl9*	uncharacterized proteinprotein C630.12	HP-dominance-Z58

670439346	GRMZM2G057865	9	123231806	123234930		*qHkl9*	probable small nuclear ribonucleoprotein G	conserved
670441164	GRMZM2G005456	9	58357290	58361458		*qHkl9*	cell division cycle and apoptosis regulator protein 1like	HP-dominance-HD568
670441318	GRMZM2G348582	9	89076551	89081864		*qHkl9*	dentin sialophosphoproteinlike	HP-dominance-HD568
819231707	GRMZM2G136838	9	26591337	26597907		*qHkl9*	kinesin like protein 2	LP-dominance-HD568
924183838	GRMZM2G040689	9	95479489	95481378		*qHkl9*	uncharacterized protein	LP-dominance-Z58
948284257	GRMZM2G042080	9	25828876	25831557		*qHkl9*	superoxide dismutase	LP-dominance-HD568
949474699	GRMZM2G022558	9	87869349	87871717		*qHkl9*	fatty acid elongase 1	conserved
162462458	GRMZM2G109472	8	157653408	157663852		*qHkt8*	uncharacterized protein	conserved
212721118	GRMZM2G133959	8	156949383	156953007		*qHkt8*	uncharacterized protein	under-dominance
226499906	GRMZM2G133885	8	156916728	156924245		*qHkt8*	uncharacterized protein	
226532126	GRMZM2G180988	8	157629081	157639477		*qHkt8*	uncharacterized protein	over-dominance
670427371	GRMZM5G838388	8	156306776	156308706		*qHkt8*	uncharacterized protein	LP-dominance-Z58
670432638	GRMZM2G133952	8	156941586	156948706		*qHkt8*	DNA mismatch repair protein MLH1	under-dominance
670432649	GRMZM2G077776	8	157262589	157264307		*qHkt8*	protein ASPARTIC PROTEASE IN GUARD CEL2 like	LP-dominance-HD568
670432658	GRMZM2G319130	8	157520383	157528514		*qHkt8*	uncharacterized protein	HP-dominance-Z58
676260273	GRMZM2G133926	8	156926212	156935667		*qHkt8*	serine/argininerich splicing	HP-dominance-HD568

						factor SR45like	
226491962	AC215244.3_FG002	4	193913422	193938699	*qHkw4*	uncharacterized protein	LP-dominance-Z58
226495885	GRMZM2G004699	4	191756828	191759110	*qHkw4*	uncharacterized protein	under-dominance
226497512	GRMZM2G117614	4	192128276	192133408	*qHkw4*	uncharacterized protein	over-dominance
226501888	GRMZM2G016805	4	193529418	193532950	*qHkw4*	uncharacterized protein	
293332531	AC190609.3_FG004	4	194583416	194589029	*qHkw4*	uncharacterized protein	HP-dominance-Z58
293334027	GRMZM2G149150	4	194939730	194943802	*qHkw4*	uncharacterized protein	HP-dominance-Z58
293336836	GRMZM5G821551	4	193324898	193329134	*qHkw4*	uncharacterized protein	LP-dominance-Z58

[a] QTL, q + planting density abbreviation + trait abbreviation + chromosome number + QTL number, e.g., qLkl1-2,corresponds to the second QTL for kernel length on chromosome 1 under low planting density.

[b] LPD, low planting density; MPD, Medium planting density; HPD, high planting density.
